# Second-Generation Radiofrequency and Targeted Therapeutic Exercise for Stress Urinary Incontinence Due to Urethral Hypermobility: A Study Protocol

**DOI:** 10.3390/healthcare14121616

**Published:** 2026-06-09

**Authors:** José P. Traña-Serrano, Cristina Orts-Ruiz, Sergio Montero-Navarro, Andrés Zamora-Streber, María José Ramírez Rivera, Oscar Garita Redondo, Francisco J. Molina-Payá, Laura Fluxa-Juan, Jesús Sánchez-Más, Cristina Salar-Andreu

**Affiliations:** 1 Clínica Traña, Nunciatura 68A, Mata Redonda, San José 10203, Costa Rica; josetrana@clinicatrana.com (J.P.T.-S.); azamora2610@gmail.com (A.Z.-S.); mari95ramirez@hotmail.com (M.J.R.R.); oskargare@gmail.com (O.G.R.); 2 Physical Therapy Department, School of Health Sciences, CEU-Cardenal Herrera University, CEU Universities, Plaza Reyes Católicos, 19, 03204 Elche, Spain; cristina.orts@uchceu.es (C.O.-R.); sergio.montero@uchceu.es (S.M.-N.); francisco.molina@uchceu.es (F.J.M.-P.); lfluxa.el@uchceu.es (L.F.-J.); cristina.salar@uchceu.es (C.S.-A.); 3 Biomedical Sciences Department, Health Sciences Faculty, CEU-Cardenal Herrera University, CEU Universities, Plaza Reyes Católicos, 19, 03204 Elche, Spain

**Keywords:** stress urinary incontinence, radiofrequency therapy, pelvic floor muscle training, urethral hypermobility, functional ultrasound, quality of life, study protocol

## Abstract

Background: Stress urinary incontinence (SUI) is defined as involuntary urine loss during activities that increase intra-abdominal pressure. It is highly prevalent among women and significantly affects physical, emotional, and social well-being. Pelvic floor muscle training (PFMT) is the gold-standard conservative therapy. Second-generation radiofrequency (RF) therapy has shown promise as an alternative. It stimulates collagen synthesis and promotes tissue remodeling. This study will compare the effects of PFMT, RF, and their combination on pelvic floor function, urethral stability, and health-related quality of life (HRQoL) in women with SUI due to urethral hypermobility. Methods/Design: This will be a single-blinded, three-arm, randomized controlled trial conducted at Clínica Traña (San José, Costa Rica). Women aged ≥18 years with clinically confirmed SUI and a retrovesical (β) angle ≥ 140° during the Valsalva maneuver on functional transperineal ultrasound will be randomized (1:1:1) to PFMT (16 weeks, twice-weekly supervised sessions), RF (5 weekly sessions using Capenergy^®^ C500 Urogyne), or combined RF + PFMT (39 per arm; total N = 117 accounting for 30% attrition). The primary outcome is the change from baseline in pelvic floor muscle strength at 12 months post-intervention, measured by the modified Oxford scale and vaginal manometry. Secondary outcomes will include urethral stability (retrovesical β angle and bladder neck descent on ultrasound), incontinence severity (Sandvik Severity Index), and HRQoL (ICIQ-UI SF and King’s Health Questionnaire). All outcomes will be assessed at baseline, immediately post-intervention, 15 days, 3 months, 6 months, and 12 months follow-up. Assessments will be performed by blinded evaluators. Analysis will follow intention-to-treat principles using repeated-measures ANOVA or non-parametric equivalents (SPSS v.29; *p* < 0.05). The trial was prospectively registered on ClinicalTrials.gov (NCT07095283, registered on 24 July 2025), prior to the recruitment of the first participant. Expected outcomes: This study will provide comparative effectiveness data on whether the addition of RF to PFMT offers additional benefits over PFMT alone in the management of SUI.

## 1. Introduction

Stress urinary incontinence (SUI) is defined by the International Continence Society (ICS) as the involuntary leakage of urine on effort or physical exertion, or on sneezing or coughing [1,2]. This protocol fully adheres to the ICS Standardization of Terminology of Lower Urinary Tract Function [2], which provides the internationally accepted conceptual framework, standardized definitions of symptoms, signs, urodynamic observations, and conditions related to lower urinary tract dysfunction. It is the most common subtype of urinary incontinence in women under 75 years of age, affecting up to 50% of this population [1,3]. The pathophysiology of SUI involves two main mechanisms: urethral hypermobility and intrinsic sphincter deficiency, as conceptualized within the ICS framework [2]. Urethral hypermobility results from weakening or laxity of the endopelvic fascia and pubourethral ligaments, leading to excessive descent of the bladder neck and proximal urethra during increases in intra-abdominal pressure (retrovesical β angle ≥ 140° on transperineal ultrasound). This anatomical displacement disrupts the normal pressure transmission to the urethra and impairs coaptation of the urethral walls. Intrinsic sphincter deficiency, in turn, is characterized by reduced urethral closure pressure due to atrophy or damage to the striated and smooth muscle components of the urethral sphincter complex. Both mechanisms frequently coexist and are exacerbated by age-related collagen loss, multiparity, and hormonal changes, ultimately resulting in failure of the urethral continence mechanism [2]. SUI is associated with substantial deterioration in health-related quality of life (HRQoL), often comparable to or greater than that observed in chronic conditions such as diabetes or hypertension [3,4].

The socioeconomic burden of SUI is substantial. It generates high direct healthcare costs (consultations, treatments, and absorbent products), indirect costs due to productivity loss and absenteeism, and important intangible costs related to reduced QoL and social isolation. Recent estimates indicate that the annual economic burden of urinary incontinence in the European Union exceeds €69 billion, with women bearing the majority of this impact [5]. These costs, together with limited access to or reluctance toward surgical options, highlight the need to explore more effective non-invasive or minimally invasive conservative strategies.

Pelvic floor muscle training (PFMT) is the gold-standard first-line conservative therapy, supported by Level A1 evidence. Supervised PFMT has been shown to significantly reduce urine leakage, improve pelvic floor muscle strength and endurance, and enhance HRQoL in women with SUI, with cure or improvement rates ranging from 50% to over 60% in multiple randomized controlled trials and meta-analyses [6]. Benefits are generally sustained with continued practice, although long-term adherence remains a challenge. Adverse events associated with PFMT are minimal.

Second-generation radiofrequency (RF) therapy has emerged as a promising non-invasive adjunctive modality. RF delivers non-ablative capacitive electrical transfer that raises tissue temperature in a controlled manner to 40–45 °C. At this therapeutic range, immediate contraction of existing collagen fibres occurs through denaturation of the triple-helix structure, while prolonged thermal stimulation activates fibroblasts, inducing neocollagenesis, neoelastogenesis, and extracellular matrix remodeling over subsequent weeks [7,8,9]. Histological studies in animal models and human tissue have demonstrated increased collagen density and improved organization of the endopelvic fascia and periurethral connective tissue following non-ablative radiofrequency, together with enhanced local vascularisation and neuromodulatory effects that may improve tissue trophism and sphincter function [10,11]. These changes are thought to restore passive urethral support and coaptation, thereby counteracting the fascial laxity central to urethral hypermobility. Although promising, the clinical evidence for intravaginal RF in SUI remains limited compared with PFMT. Available studies, mainly small randomized trials and pilot studies, have reported significant short-term reductions in incontinence episodes and improvements in QoL, with low rates of adverse events (mainly mild transient vaginal discomfort or discharge) [12]. However, most trials have small sample sizes, short follow-up periods, and heterogeneous protocols, leaving uncertainty regarding the magnitude and duration of benefit, as well as the added value of combining RF with supervised PFMT.

Current international guidelines (AUA/SUFU 2023, EAU, ICI, and NICE) unanimously recommend intensive supervised PFMT as the initial conservative management for women with SUI, reserving surgery (primarily mid-urethral sling) for cases where conservative therapy fails or is declined [13,14]. Pharmacological options have a very limited role in pure SUI. Current EAU Guidelines on Non-neurogenic Female LUTS (2026) do not recommend RF therapy as standard conservative treatment for SUI and advise that energy-based vaginal therapies should only be offered within the context of well-regulated research studies [14].

In this context, evaluating whether the addition of second-generation RF to standard PFMT can provide superior clinical outcomes represents a relevant research question with potential clinical and public health implications.

The study protocol outlines a prospective interventional study that will evaluate the effectiveness of PFMT, RF, and their combination in improving pelvic floor function, reducing urethral hypermobility, and enhancing HRQoL in women with SUI.

## 2. Materials and Methods

### 2.1. Trial Design

This study is designed as a single-blinded, randomized controlled trial to evaluate the effectiveness of PFMT, RF therapy, and their combination in women with SUI. The trial will be conducted at Clínica Traña, San José, Costa Rica. The study will follow the guidelines set according to CONSORT [15] and the SPIRIT Statement [16] (Supplementary Material, File S1). Table 1 describes the study in detail.

This is a single-blinded randomized controlled trial. Participants and the physiotherapists delivering the interventions cannot be blinded because of the nature of the treatments. However, blinded evaluators will perform all outcome assessments, including pelvic floor evaluations, ultrasound examinations, and questionnaires. Statistical analysts will also remain blinded until the database is locked.

To maintain blinding, outcome assessors will not have access to the randomization list, will not participate in recruitment or intervention delivery, and will not be present during envelope opening. Participants will be explicitly instructed not to discuss their assigned treatment with the outcome assessors at any follow-up visit. In the event of accidental unblinding (e.g., a participant inadvertently revealing their group), the event will be documented on a specific unblinding form, including date, circumstances, and potential impact on the assessment. The principal investigator will evaluate the situation and decide whether the affected assessment should be repeated by another blinded assessor or excluded from analysis. All unblinding events will be reported in the final study publication.

Important design considerations and limitations. This is a three-active-arm pragmatic trial without a no-treatment control group or sham/placebo radiofrequency arm. The absence of a no-treatment control was chosen for ethical reasons, as supervised PFMT is the established first-line treatment for symptomatic SUI (Level A1 evidence). A credible sham RF procedure was not feasible because the device produces a perceptible heating sensation that would compromise blinding. Intervention durations also differ across arms (5 weeks for RF vs. 16 weeks for PFMT), reflecting the distinct biological mechanisms and time courses of each intervention. These design choices, while pragmatic and clinically relevant, represent the main methodological limitations of the trial and are discussed in detail in the Limitations Section.

The trial was prospectively registered on ClinicalTrials.gov (identifier: NCT07095283) on 24 July 2025, prior to the start of participant recruitment. All primary and secondary outcomes assessment timepoints reported in this protocol are consistent with those prespecified in the trial registration.

### 2.2. Study Setting and Study Population

The study will be conducted at Clínica Traña (San José, Costa Rica). Recruitment will target women who attend the clinic seeking assessment and treatment of urinary incontinence. Potential participants will be identified through the clinic’s usual communication channels (phone calls and social media inquiries used to schedule appointments). Each candidate will then be contacted by telephone by the researcher to provide preliminary study information and assess eligibility. Eligible women will be scheduled for a face-to-face evaluation visit. Upon attendance at the clinic for this assessment, participants will receive individualized information about the study, the evaluation procedures, and the intervention protocols. Those who agree to participate will complete a detailed online questionnaire regarding demographics, obstetric and gynaecological history, comorbidities, surgical history, and baseline HRQoL and incontinence questionnaires: International Consultation on Incontinence Questionnaire—Urinary Incontinence Short Form (ICIQ-UI SF), King’s Health Questionnaire and Sandvik severity index.

The study population will therefore consist of women with clinically confirmed SUI who attend the clinic for assessment and who, after being fully informed, voluntarily agree to participate.

### 2.3. Sample Size and Randomization

The sample size was calculated based on the primary outcome (change in pelvic floor muscle strength measured by vaginal manometry). Data from a previous randomized controlled trial [13] showed a mean difference of 12.4 cmH_2_O between groups at the end of treatment, with a pooled standard deviation of 18.2 cmH_2_O. Assuming a conservative effect size (Cohen’s f = 0.25) for the three-group comparison, an alpha level of 0.05, a correlation among repeated measures of r = 0.5, and a nonsphericity correction ε = 1 for the three-group comparison, an alpha level of 0.05, and a statistical power of 80% (1 − β = 0.80), the required sample size was estimated at 87 participants (29 per group) using G*Power software (version 3.1) for a repeated-measures ANOVA (within-between interaction). Accounting for an anticipated 30% attrition rate during the 12-month follow-up, the final sample size was increased to 117 participants (39 per group).

Randomization will be executed using SAS 9.4 in a 1:1:1 allocation ratio, with concealment achieved through sequentially numbered, opaque envelopes. The allocation process will be managed by an investigator (J.S.-M.) who remains blinded to the outcome evaluations. Blinding of participants to their assigned intervention is not feasible.

### 2.4. Eligibility Criteria

The inclusion criteria comprise adult women experiencing any degree of urine leakage assessed by ICIQ-UI SF and urethral hypermobility, defined as a retrovesical (β) angle ≥ 140° during the Valsalva maneuver pelvic floor ultrasonography [17]. The retrovesical (β) angle is the angle between the posterior wall of the bladder and the longitudinal axis of the urethra.

Exclusion criteria include concurrent pelvic floor dysfunctions other than SUI, current pregnancy, inability to visualize anatomical structures via ultrasound, prior PFMT or RF within the past 12 months, recent vaginal or systemic estrogen therapy, laser therapy within the past 6 months, pacemakers, heart failure, metabolic or neurological disorders, cognitive deficits, cutaneous lesions, active urinary or vaginal infection, or previous pelvic surgery. This criterion was included because prior pelvic surgery (e.g., hysterectomy, mid-urethral sling, or prolapse repair) can significantly alter pelvic floor anatomy, tissue elasticity, and urethral support mechanisms. Including these participants could introduce substantial heterogeneity in the response to RF and PFMT, thereby confounding the interpretation of the results in this initial randomized trial focused on primary urethral hypermobility. Participants will provide written informed consent prior to enrollment.

### 2.5. Recruitment

Participants will be recruited during the enrollment period between February and May 2026 from Clínica Traña (San José, Costa Rica). This recruitment and enrollment window corresponds to the enrollment phase shown in Table 1. Potential participants will be identified by the research team through systematic screening of women attending Clínica Traña for assessment or treatment of urinary incontinence. Identification will occur via the clinic’s routine communication channels (telephone calls and social media inquiries for appointment scheduling). Each candidate will be contacted individually by telephone by a member of the research team to provide preliminary study information, conduct initial eligibility screening, and, if appropriate, schedule a face-to-face evaluation visit. This individualized dissemination of information ensures that women receive personalized explanations in a private setting before deciding on participation. Individuals who express interest but do not meet the eligibility criteria will receive an initial assessment, be informed about the status of their pelvic function, and be given therapeutic recommendations; however, they will not be included in the study.

### 2.6. Allocation

The randomization sequence will be generated by an independent statistician using SAS 9.4 software (SAS Institute, Cary, NC, USA) in a 1:1:1 allocation ratio, with randomly permuted block sizes of 3 and 6 to ensure balance across the three arms. The allocation list will be concealed from all investigators involved in recruitment and outcome assessment.

Numerical identifiers corresponding to the study arms (1 = RF; 2 = PFMT; 3 = RF + PFMT) will be placed inside sequentially numbered, sealed, opaque, tamper-evident envelopes. The envelopes will be prepared and sealed by the independent statistician and stored in a locked cabinet at Clínica Traña under the custody of a research coordinator not involved in participant enrollment or outcome assessment.

Envelopes will be opened sequentially by the enrolling investigator (J.P.T.-S.) only after the participant has provided written informed consent and completed all baseline assessments. The allocation will be revealed to the participant immediately after the envelope is opened by the investigator. Both the participant and the investigator will sign a form documenting the envelope number, opening date, and assigned group. The opened envelopes will be stored separately for audit purposes.

### 2.7. Initial Assessment

On the day of the initial consultation, the first contact with potential participants will always be conducted by the same investigator (J.P.T.-S.), who will provide detailed information about the study and deliver the participant information sheet along with the informed consent form.

Upon acceptance of participation in the study, participants will complete an online questionnaire (Google Forms) regarding sociodemographic data, obstetric and gynaecological history, comorbidities, and surgical history. During the face-to-face initial consultation, height and weight will be objectively measured using a stadiometer and calibrated scale to calculate body mass index (BMI). This questionnaire will also include the Sandvik Severity Index, ICIQ-UI SF, and King’s Health Questionnaire.

This initial consultation will be supplemented by an ultrasound assessment, during which various characteristics will be evaluated, such as pelvic floor muscle strength and endurance, vaginal wall instability, urethral hypermobility, rectal instability, and the presence of prolapses at rest or during the Valsalva maneuver (which involves a forced expiration against a closed glottis with contraction of the diaphragm and abdominal wall).

After the random allocation described above, participants will be informed of the assigned group and the treatment schedule by means of an opaque envelope. Participants will attend the program on the dates indicated in the calendar and will be given their therapist’s contact telephone number so that they can ask any questions they may have about the intervention.

### 2.8. Interventions

*1.*  *Group 1. RF-based treatment*

**Figure 1:**
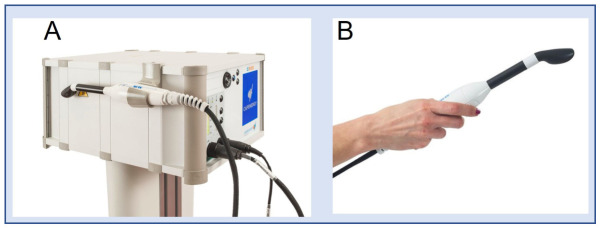
Capenergy C500 Medical Tecar Therapy system and intravaginal electrode: (**A**) Capenergy C500 medical device unit; (**B**) detailed view of the MJS intravaginal electrode used for RF treatment application.

The proposed RF therapy will employ capacitive electrical transfer using the Capenergy^®^ device (model C500 Urogyne; Capenergy Medical, Barcelona, Spain), specifically designed for urogynaecological dysfunctions, with tissue temperature regulated by an integrated sensor, three selectable frequencies (0.8 MHz, 1 MHz, and 1.2 MHz) to target different tissue depths, and a maximum power of 310 W. The system includes an active capacitive electrode placed intravaginally with a probe cover and water-soluble gel, and a dispersive return electrode positioned on the lumbosacral region (Figure 1).

The protocol delivers controlled thermal energy (target temperature of 41 °C at 1 MHz frequency, with a total energy delivery of 75 kJ per session) to the vaginal wall and endopelvic fascia. Although this protocol was originally adapted from treatments used in genitourinary syndrome of menopause, its application in SUI due to urethral hypermobility is supported by distinct pathophysiological mechanisms. In women with SUI, there is frequently a reduction in collagen content and tensile strength of the endopelvic fascia and periurethral connective tissues, which compromises urethral support and coaptation during increases in intra-abdominal pressure. Non-ablative RF induces immediate contraction of existing collagen fibres and stimulates long-term fibroblast activation, promoting neocollagenesis, neoelastogenesis, and progressive tissue remodeling. These histological changes enhance the passive supportive capacity of the anterior vaginal wall and periurethral structures, thereby improving urethral stability and closure function. This rationale is supported by clinical studies demonstrating significant improvements in SUI symptoms, reduction in urine leakage, and enhanced pelvic floor support following intravaginal non-ablative RF [9,18,19].

The physiotherapist will place participants in the dorsal lithotomy position with flexed and supported legs. Once the target temperature is achieved, the physiotherapist will perform controlled semi-circular movements with the active intravaginal capacitive electrode for 2 min on the anterior vaginal wall and 4 min on the posterior vaginal wall. The longer application time on the posterior vaginal wall is intended to promote collagen remodeling and strengthening of the fascial and muscular structures that provide indirect support to the urethra and bladder neck. The total duration per session is approximately 6 min of active treatment, with 5 sessions spaced 7 days apart (spanning 4 weeks overall). Participants will be instructed to contact the research team immediately if they experience any discomfort or notice changes in vaginal discharge during or after treatment.

A detailed step-by-step protocol of the RF intervention is provided in Supplementary Material (File S2).

*2.*  *Group 2. PFMT-Based Treatment*

PFMT will consist of a 16-week structured program, with two 45 min sessions per week in small groups of eight [20]. Exercises target isolated pelvic floor contractions and synergistic activation with the core musculature, incorporating static and dynamic postures, unstable surfaces, progressive loads, and hypopressive techniques. Each session progresses in difficulty, maintaining motivation and adherence. A detailed description of the PFMT program can be found in Supplementary Material (File S3). The individual pictured in the physical exercise program description has provided written informed consent to publish their image alongside the manuscript.

*3.*  *Group 3. RF + PFMT*

Participants assigned to the combined group will receive the full 16-week PFMT program. RF therapy (5 weekly sessions) will be administered during the last 5 weeks of the PFMT program (weeks 12–16).

The duration and cumulative dose of the interventions differ across study arms. RF therapy consists of 5 weekly sessions (approximately 5 weeks), while PFMT is delivered over 16 weeks. In the combined arm, RF is administered during weeks 12–16 of the PFMT program. This pragmatic design reflects the distinct biological time courses of each intervention (rapid thermal collagen remodeling with RF versus progressive neuromuscular adaptation with supervised PFMT) and mirrors current clinical practice, in which adjunctive radiofrequency is frequently added in the final phase of an exercise-based program.

### 2.9. Outcomes

The primary outcome of this study will be the change from baseline in pelvic floor muscle strength at 12 months post-intervention, measured by the combination of the modified Oxford scale (qualitative assessment) and vaginal manometry (maximum voluntary contraction pressure in cmH_2_O using the PHENIX LIBERTY system). This objective physiological measure was selected as primary because it provides a direct, reproducible assessment of the mechanistic effect of the interventions on pelvic floor support and is less prone to expectation bias.

To ensure a strong patient-centered focus, the following clinically meaningful outcomes will be evaluated as key secondary outcomes: incontinence severity (Sandvik Severity Index), symptom impact and HRQoL (ICIQ-UI SF and King’s Health Questionnaire). These patient-reported outcomes directly capture the real-world benefit experienced by women with SUI, including frequency and amount of leakage, limitations in daily activities, social participation, emotional well-being, and overall quality of life.

Secondary outcomes will also include urethral stability (retrovesical β angle and bladder neck descent on transperineal ultrasound). All outcomes will be assessed at baseline, immediately post-intervention, 15 days, 3 months, 6 months, and 12 months follow-up.

*1.*  *Assessment Criteria*

Ultrasound evaluation [17,21,22]: For the ultrasound assessment, participants will be instructed to arrive with a filled bladder to facilitate enhanced imaging quality and to evaluate the degree of support provided by the vaginal walls in response to the counterpressure exerted by the bladder. Perineal imaging will be conducted utilizing a MyLab X8 ultrasound system fitted with a 3.5–6 MHz convex probe (Esaote, Florence, Italy). The procedure will be carried out in the dorsal lithotomy position, involving hip flexion with mild abduction and positioning of the heels near the buttocks, or in an upright stance when required. The probe, encased in a protective sheath, will be oriented longitudinally along the midline perineum to yield a midsagittal plane, enabling visualization of the pubic symphysis, urethra, and bladder neck.

The posterior β angle will be quantified at rest and during the Valsalva maneuver. The retrovesical (β) angle ≥ 140° during maximal Valsalva was used as the ultrasound criterion for urethral hypermobility and study inclusion. Subsequently, a 5.0 MHz transducer, positioned identically and with unchanged bladder volume, will be utilized to assess bladder neck funneling both in the absence and presence of the Valsalva maneuver. Bladder neck descent will also be documented, defined as the displacement observed during the maximal Valsalva maneuver (determined from the best of at least three attempts) relative to the inferoposterior margin of the pubic symphysis.

To minimize information bias in the study, the radiologist will be blinded to the results of the clinical examinations.

*2.*  *Assessment of Pelvic Floor Muscle Strength*

This evaluation will quantify the strength, endurance, and fatigability of the pelvic floor musculature, which plays a critical role in supporting the pelvic viscera and preserving urinary and fecal continence. Before the assessment, participants will be requested to empty their bladder to achieve the greatest possible standardization of bladder volume [23]. Subsequently, they will assume a seated posture for a 3 min rest period prior to measurement, representing double the duration necessary for sympathetic nervous system deactivation.

For the strength evaluation, participants will be placed in the lithotomy position, with the genital area and lower extremities exposed yet draped with a sheet, and directed to maintain a relaxed state [24].

Pelvic floor muscle strength will be assessed by digital palpation using the modified Oxford scale (0–5). Although the modified Oxford scale has shown moderate to good intra-rater reliability (κ = 0.69–0.86) and fair to substantial inter-rater reliability (κ = 0.33–0.81) in previous studies [24,25], its subjective nature is well recognized. To minimize variability, all assessments will be performed by the same blinded and experienced physiotherapist who has received specific training in pelvic floor muscle assessment according to ICS recommendations. The assessor has more than 5 years of clinical experience in urogynecological physiotherapy and has been previously standardized in the use of the modified Oxford scale through practical training sessions with feedback until achieving consistent scoring (intra-rater agreement > 0.80). This qualitative assessment provides information on contraction quality, correct lifting direction, symmetry, and endurance [9,26,27].

Muscle strength will also be evaluated by transperineal ultrasound, which does not directly measure force generation but rather displacement of pelvic structures as a proxy for pelvic floor muscle contractile function and coordination. In the midsagittal plane, the following displacement measurements will be obtained during maximal voluntary pelvic floor muscle contraction (best of three attempts):

- Bladder neck displacement (cranio-caudal movement relative to the inferoposterior margin of the pubic symphysis).
- Anteroposterior displacement of the levator hiatus.
- Reduction in levator hiatal area.

These ultrasound displacement parameters serve as an indirect indicator of pelvic floor muscle function but are recognized as a poor direct proxy for muscle strength, particularly because even a weak muscle may achieve full range of motion depending on position and gravity. Therefore, ultrasound findings will be interpreted as complementary to, and not a substitute for, the quantitative manometric assessment.

Muscle strength and endurance will further be evaluated utilizing a perineometer equipped with an inflatable vaginal probe interfaced to the PHENIX LIBERTY neuromuscular stimulation and manometry apparatus (Electronic Concept Lignon Innovation, Montpellier, France) [28]. The probe, which is air-inflated and encased in a latex sheath lubricated with gel, will be linked to the Phenix biofeedback system for data acquisition. Pelvic floor pressure signals will be captured, encompassing basal tone and the peak pressure maintained over a 10 s interval, with triplicate measurements obtained. The average of these three values will be computed.

During the manometric measurement, participants will be given the following clear instructions: “Please squeeze and lift your pelvic floor muscles as strongly as you can, as if you were trying to stop the flow of urine and hold it for as long as possible.” Verbal encouragement will be provided as needed.

The combined use of digital palpation and manometry provides both a qualitative clinical assessment and a reliable quantitative measure of pelvic floor muscle function. Although intravaginal manometry is minimally invasive, it is well tolerated by participants and routinely used in urogynecological research when accurate and objective data on pelvic floor muscle strength and endurance are required as primary outcomes.

*3.*  *Quality of Life and Severity of SUI*

International Consultation on Incontinence Questionnaire—Urinary Incontinence Short Form (ICIQ-UI SF) [4]: This self-administered questionnaire evaluates the frequency, amount, and impact of urinary leakage on QoL, generating a total score from 0 to 21, with higher scores indicating greater impact. The ICIQ-UI SF has been validated as a brief and robust measure of UI symptoms and their effect on QoL across diverse clinical and research settings.

Sandvik Severity Scale (Incontinence Severity Index) [29]: The Sandvik scale classifies SUI based on two questions addressing frequency and amount of urine loss. Scores are multiplied to produce a total index, categorizing incontinence from mild to very severe, with higher values reflecting greater severity. This index has been widely used in both epidemiological and clinical studies and validated against objective measures such as pad tests.

King’s Health Questionnaire (KHQ) [30]: The KHQ is a self-administered instrument specifically designed to assess HRQoL in women with UI. It consists of 21 items distributed across 9 dimensions: perception of overall health (1 item), impact of UI (1 item), limitations in daily activities (2 items), physical limitations (2 items), social limitations (2 items), personal relationships (3 items), emotions (3 items), sleep/energy (2 items), and severity of UI (5 items). The score range for each dimension is from 0 (lowest impact of UI and therefore best QoL) to 100 (highest impact, worst QoL). This questionnaire provides an overall value for the HRQoL of the patient with UI (Overall Score-OS) and another specific value for each dimension on a scale with the following range: 0: Best possible QoL–100: Worst possible QoL.

### 2.10. Baseline and Follow-Up Assessment

Following informed consent, participants will undergo the previously described baseline evaluation during their initial visit. This includes an anamnesis via an ad hoc questionnaire to capture sociodemographic characteristics and SUI diagnosis/treatment details, completion of the King’s Health Questionnaire, ICIQ-UI SF, Sandvik Severity Index, Oxford scale, and ultrasound assessment, followed by a physical examination comprising pelvic floor muscle strength evaluation through biofeedback. Questionnaires will be self-administered by participants and collected at the conclusion of the assessment.

Participants will be monitored at four post-intervention time points: the initial follow-up (15 days post-intervention), the second follow-up (three months post-intervention), the third follow-up (six months post-intervention), and the fourth follow-up (twelve months post-intervention). Identical assessment protocols to those utilized in the baseline evaluation will be applied during each subsequent follow-up.

### 2.11. Data Management

All study data will be managed according to the principles of the European General Data Protection Regulation (GDPR, Regulation (EU) 2016/679) and Spanish data protection regulations.

Personal data will be collected through Google Forms for the initial online questionnaire. Immediately after collection, limited personal identifiers (name, surname, email address, telephone number, and national identification number) are collected solely for the purposes of informed consent and follow-up contact and will be pseudonymized by assigning a unique study code (e.g., SUI-001). The key linking the study code to the participant’s identity will be stored separately in a password-protected file accessible only to the principal investigator (J.P.T.-S.) and the data manager.

Data entry and storage will be performed in a secure electronic database hosted at Universidad CEU Cardenal Herrera. Access to the database will be role-based and strictly controlled:

- Only the data manager and the statistician (J.S.-M.) will have full access for data cleaning and analysis.
- Outcome assessors and intervention therapists will have no access to the database.
- The principal investigator will have read-only access for monitoring purposes.

All data transfers will be performed through encrypted channels. Daily incremental backups and weekly full backups will be automatically performed and stored on secure servers with 256-bit AES encryption. An audit trail will record all accesses, modifications, and deletions within the database.

Study data will be retained for a minimum of 5 years after study completion or publication of the main results, in accordance with institutional and regulatory requirements. At the end of the retention period, all identifiable data will be permanently deleted, and the linkage key will be destroyed.

### 2.12. Adverse Events and Safety Monitoring

Adverse events will be classified according to severity using the Common Terminology Criteria for Adverse Events (CTCAE v5.0): Grade 1 (mild), Grade 2 (moderate), Grade 3 (severe), Grade 4 (life-threatening), and Grade 5 (death). Causality will be assessed by the principal investigator as definitely related, probably related, possibly related, unlikely related, or unrelated to the study intervention.

Systematic detection of AEs will occur through:

- Active questioning by the physiotherapist at each RF session and PFMT session using a standardized checklist.
- Open-ended questioning at every follow-up visit (“Have you experienced any health problems or symptoms since the last visit?”).
- Review of medical records when clinically indicated.

All AEs will be documented on a standardized adverse event form, including date of onset, duration, severity, causality, action taken, and outcome. Serious adverse events will be reported to the Ethics Committee of Universidad CEU Cardenal Herrera within 24 h of the investigator becoming aware of the event. All AEs will be monitored from the start of the intervention until the final 12-month follow-up visit.

PFMT is generally considered a safe intervention with a very low incidence of adverse effects when properly supervised. For RF therapy, the most commonly expected AEs are mild transient vaginal discomfort, increased discharge, or spotting. The RF procedure will be immediately stopped if a participant reports discomfort ≥ 4 on the Visual Analogue Scale (VAS) or if any signs of vaginal trauma or infection [31,32]. In these instances, suitable medical care will be administered as required.

Participants exhibiting less than 80% attendance in RF or PFMT sessions will be considered non-adherent to the intervention protocol. In such cases, the active intervention will be discontinued; however, these participants will continue to be invited to attend all scheduled follow-up assessments. All randomized individuals, including those who withdraw from the intervention or demonstrate suboptimal compliance, will be included in the final data analysis using an intention-to-treat approach.

### 2.13. Data Analysis

Data will be examined utilizing an intention-to-treat framework. Normality of distribution will be evaluated via the Kolmogorov–Smirnov test and visual inspection (Q-Q plots and histograms).

The primary longitudinal analysis for group-by-time interactions will be performed using linear mixed-effects models (LMM) with group (RF, PFMT, combined), time, and the group × time interaction as fixed effects, participant as a random intercept, and (where model fit improves significantly) a random slope for time. An unstructured or autoregressive covariance structure will be selected based on the lowest Akaike Information Criterion (AIC). Where appropriate for binary or ordinal outcomes, generalised linear mixed models (GLMM) will be employed. This approach is preferred as it is more robust to missing data, accommodates unequal assessment intervals, and accounts for within-subject correlation more efficiently than traditional repeated-measures ANOVA.

As a sensitivity analysis, the results obtained from repeated-measures ANOVA (or non-parametric Friedman test when assumptions are not met) will also be presented. Where sphericity is violated, the Greenhouse–Geisser correction will be applied.

The primary analysis will focus on the group-by-time interaction for the primary outcome (change in pelvic floor muscle strength from baseline to 12 months post-intervention). To control the overall type I error rate due to multiple comparisons (multiple outcomes and multiple time points), the following strategy will be applied:

- For the primary outcome at the primary time point (12 months), statistical significance will be set at *p* < 0.05 without adjustment.
- For the primary outcome at the other time points (15 days, 3 months, and 6 months) and for all secondary outcomes across all time points, the significance level will be adjusted using the Bonferroni correction (α = 0.05 divided by the number of comparisons).
- Secondary outcomes will be interpreted cautiously, considering both adjusted and unadjusted *p*-values, together with effect sizes (Cohen’s d or partial eta squared) and clinical relevance. Where appropriate, hierarchical testing procedures will be applied to preserve statistical power while maintaining rigor.

Missing data will be handled using multiple imputation by chained equations (MICE) under the missing-at-random (MAR) assumption. The number of imputations will be set at 20. A complete-case sensitivity analysis will also be performed to assess the robustness of the results.

All analyses will be performed with SPSS version 29 (SPSS Inc., Chicago, IL, USA), with statistical significance set at *p* < 0.05 after correction.

### 2.14. Trial Status

On 1 February 2026, informational discussions and participant recruitment for the study will commence. Recruitment will conclude on 15 May 2026. The intervention will begin in March 2026 (immediately after the first participants are randomized) and will run until August 2026, with data collection anticipated to be completed by 2027. The final results are expected to be available in June 2027.

### 2.15. Post-Trial Care and Compensation

Given the non-invasive nature of the interventions (PFMT and second-generation intravaginal RF), no specific post-trial care is required beyond routine clinical follow-up. Participants who wish to continue treatment after the end of the study period will be offered continued care at Clínica Traña according to standard clinical practice and at their own expense or through their usual healthcare coverage.

No additional compensation will be provided to participants for taking part in the study, as the interventions are considered low-risk and are provided free of charge during the trial period. All participants are covered by the standard liability insurance of Universidad CEU Cardenal Herrera and Clínica Traña. In the unlikely event of any study-related injury, participants will receive appropriate medical care according to current Spanish regulations. No additional compensation beyond standard clinical care will be offered.

## 3. Discussion

The anticipated findings of this study are expected to demonstrate the effects of RF therapy, PFMT, and their combined application in women with SUI associated with urethral hypermobility, defined as a retrovesical (β) angle ≥ 140° during the Valsalva maneuver on functional transperineal ultrasound. Patient-centered outcomes, including incontinence severity and HRQoL, will be given equal interpretive weight when translating the trial results into clinical practice.

From a biological perspective, the combination offers plausible synergistic mechanisms. RF delivers controlled thermal energy that induces immediate contraction of collagen fibres and stimulates long-term neocollagenesis, neoelastogenesis, and remodeling of the endopelvic fascia and anterior vaginal wall. These structural changes are expected to improve passive urethral support and coaptation. In parallel, targeted PFMT promotes neuromuscular adaptations, including increased muscle strength, endurance, coordination, and automatic activation during increases in intra-abdominal pressure. The combined intervention may therefore address both the passive connective tissue component and the active muscular component of urethral closure, potentially offering greater improvement than either modality alone.

We hypothesize that both interventions, particularly when combined, will lead to significant improvements in urinary control, pelvic floor function, urethral stability, and HRQoL, likely due to these complementary structural and neuromuscular effects.

Supervised PFMT remains the first-line conservative treatment for SUI, supported by Level A1 evidence, with documented cure or improvement rates of 50–60% in multiple randomized trials. Current EAU Guidelines on Non-neurogenic Female LUTS (2026) strongly recommend intensive supervised PFMT as the initial conservative management for women with SUI and do not endorse energy-based therapies such as non-ablative radiofrequency outside well-regulated research protocols due to limited high-quality long-term evidence. In this context, RF is not positioned as an alternative to PFMT but as a potential adjunctive therapy that could enhance its effectiveness, particularly in women with suboptimal response to exercise alone or those seeking faster or more sustained results.

Clinically, these findings could promote early physiotherapy interventions and reduce reliance on surgical approaches. If superiority of the combined approach is demonstrated, the results could have relevant implications for conservative management algorithms, reduction in surgical referrals, and integration of RF into standard rehabilitation pathways.

Beyond clinical efficacy, the results could carry important healthcare and economic implications. SUI generates a substantial socioeconomic burden, including direct costs (consultations, absorbent products, and surgery), indirect costs (productivity loss and absenteeism), and reduced QoL. A more effective non-invasive combined intervention could improve treatment adherence, decrease long-term reliance on absorbent products, and potentially reduce the need for surgical procedures, thereby optimizing resource utilization and improving cost-effectiveness within public and private healthcare systems.

In summary, this study will evaluate whether the combination of RF and targeted PFMT may provide additional benefits over PFMT alone. The findings could provide a scientific foundation for broader clinical implementation of combined RF and PFMT in the evidence-based management of SUI.

### Limitations of the Study Design

The study presents several methodological limitations that should be acknowledged. Due to the nature of the interventions, double-blinding is not feasible. Additionally, the duration of the interventions differs between groups (5 weeks for RF versus 16 weeks for PFMT), which may influence participant expectations and introduce potential placebo effects. The study does not include a no-treatment control group or a sham/placebo radiofrequency arm. This decision was made for ethical reasons, as all participants presented with clinically significant symptomatic SUI and withholding active treatment would not be justifiable given the established efficacy of supervised PFMT, the current first-line conservative therapy (Level A1 evidence). Furthermore, developing a credible sham procedure for intravaginal radiofrequency is particularly challenging because the device produces a perceptible thermal sensation, which could compromise participant blinding and introduce significant expectation bias. Instead, this three-active-arm pragmatic design allows direct comparison between the gold-standard intervention (PFMT), RF monotherapy, and their combination. Although this design does not allow estimation of absolute treatment effects relative to no intervention, it addresses the more relevant clinical question of comparative effectiveness and the potential added benefit of RF when combined with PFMT.

The primary challenge regarding internal validity is ensuring adequate participant adherence to the PFMT protocol and maintaining consistency in training intensity. To address these issues, ongoing supervision will be provided, participants’ perceived exertion will be regularly assessed, and strategies to maximize adherence will be implemented. Potential attrition bias and missing data will be handled through multiple imputation and sensitivity analyses (including complete-case analysis), with all the results evaluated using an intention-to-treat approach. Despite these limitations, the inclusion of a 12-month follow-up period, blinded outcome assessment, and rigorous statistical methods will help strengthen the internal validity and robustness of the findings.

The three intervention arms involve different treatment durations and cumulative doses (16 weeks of PFMT versus 5 weeks of RF). This pragmatic design may introduce differential expectation effects, as participants in the longer-duration arms (PFMT and combined therapy) might perceive their intervention as more intensive or credible compared with the shorter RF-only arm. This design reflects the distinct biological mechanisms and time courses of action of each intervention and mirrors clinical practice, where adjunctive RF is often added toward the end of an exercise program. Although complete blinding of participants is not feasible, all outcome assessments are performed by fully blinded evaluators, and the primary analysis relies on the group-by-time interaction in repeated-measures models up to 12 months post-intervention. This longitudinal approach minimizes bias arising from acute exposure differences. Effect sizes will be reported to support clinical interpretation.

## 4. Conclusions

This study protocol describes a single-blinded, three-arm randomized controlled trial that will evaluate the comparative effectiveness of supervised PFMT, RF therapy, and their combination in women with SUI due to urethral hypermobility. By incorporating objective functional ultrasound assessment of urethral stability, quantitative manometry of pelvic floor muscle strength, and validated patient-reported outcomes, the trial aims to generate high-quality evidence on the comparative and potential additive effects of these non-invasive interventions over a 12-month follow-up period.

The results of this study are expected to clarify whether the addition of RF to standard PFMT provides superior improvements in pelvic floor function, urethral support, incontinence severity, and HRQoL compared with PFMT alone. The findings will also contribute to addressing current evidence gaps regarding long-term outcomes and the potential synergistic mechanisms of thermal tissue remodeling and neuromuscular training in the conservative management of SUI.

## Figures and Tables

**Table 1:** Description of the stages of the study. Participant timeline: Schedule of enrollment, interventions, and assessments.

Study Period	Enrollment	Intervention	Post-Intervention Follow-Up			
Timepoint	Feb–May 2026	Mar–Aug 2026	15 d	3 mo	6 mo	12 mo
ENROLLMENT						
Eligibility screen	X					
Informed consent	X					
Baseline assessments *	X					
Randomization	X					
INTERVENTIONS						
RF		X				
PFMT		X ^†^				
Combined therapy (RF + PFMT)		X ^†^				
ASSESSMENTS						
Physical examination	X		X	X	X	X
Functional transperineal ultrasound	X		X	X	X	X
Pelvic floor muscle strength (Oxford scale + manometry)	X		X	X	X	X
ICIQ-UI SF, King’s Health Questionnaire, Sandvik Severity Index	X		X	X	X	X

* Baseline assessments include: physical examination, functional ultrasound, pelvic floor muscle strength assessment, and all patient-reported outcome questionnaires (ICIQ-UI SF, King’s Health Questionnaire, and Sandvik Severity Index). ^†^ PFMT is delivered over 16 weeks (twice weekly). In the combined arm, RF is administered during weeks 12–16 of the PFMT program. ICIQ-UI SF: International Consultation on Incontinence Questionnaire—Urinary Incontinence Short-Form.

## Data Availability

The data resulting from the trial will be available on Zenodo, an open-access repository developed under the European OpenAIRE program and operated by CERN.

## Supplementary Materials

The following supporting information can be downloaded at https://www.mdpi.com/article/10.3390/healthcare14121616/s1, File S1. SPIRIT checklist study protocol. File S2. RF Program. File S3. PFMT Program.
