# Supplementary material for: Second-Generation Radiofrequency and Targeted Therapeutic Exercise for Stress Urinary Incontinence Due to Urethral Hypermobility: A Study Protocol
Source: Healthcare (Basel). 2026 Jun 9;14(12):1616. doi: 10.3390/healthcare14121616 (PMC13299678; doi:10.3390/healthcare14121616)
Supplement: Supplementary file 1 [file healthcare-14-01616-s001.zip › healthcare-4296637-supplementary.pdf]

S1 File. SPIRIT Checklist Study Protocol

SPIRIT 2025 checklist of items to address in a randomized trial protocol*

Section / Topic	No	SPIRIT 2025 checklist item description	Reported on page no.
**Administrative information**			
Title and structured summary	1a	Title stating the trial design, population, and interventions, with identification as a protocol	1
	1b	Structured summary of trial design and methods, including items from the World Health Organization Trial Registration Data Set	1
Protocol version	2	Version date and identifier	1
Roles and responsibilities	3a	Names, affiliations, and roles of protocol contributors	1 / 14
	3b	Name and contact information for the trial sponsor	14
	3c	Role of trial sponsor and funders in design, conduct, analysis, and reporting of trial; including any authority over these activities	14
	3d	Composition, roles, and responsibilities of the coordinating site, steering committee, endpoint adjudication committee, data management team, and other individuals or groups overseeing the trial, if applicable	
**Open science**			
Trial registration	4	Name of trial registry, identifying number (with URL), and date of registration. If not yet registered, name of intended registry	1 / 3
Protocol and statistical analysis plan	5	Where the trial protocol and statistical analysis plan can be accessed	
Data sharing	6	Where and how the individual de-identified participant data (including data dictionary), statistical code, and any other materials will be accessible	15
Funding and conflicts of interest	7a	Sources of funding and other support (e.g., supply of drugs)	15
	7b	Financial and other conflicts of interest for principal investigators and steering committee members	15
Dissemination policy	8	Plans to communicate trial results to participants, healthcare professionals, the public, and other relevant groups (e.g., reporting in trial registry, plain language summary, publication)	15
**Introduction**			
Background and rationale	9a	Scientific background and rationale, including summary of relevant studies (published and unpublished) examining benefits and harms for each intervention	2 / 3

	9b	Explanation for choice of comparator	2 / 3
Objectives	10	Specific objectives related to benefits and harms	3
**Methods: Patient and public involvement, trial design**			
Patient and public involvement	11	Details of, or plans for, patient or public involvement in the design, conduct, and reporting of the trial	3
Trial design	12	Description of trial design including type of trial (e.g., parallel group, crossover), allocation ratio, and framework (e.g., superiority, equivalence, non-inferiority, exploratory)	3 / 4
**Methods: Participants, interventions, and outcomes**			
Trial setting	13	Settings (e.g., community, hospital) and locations (e.g., countries, sites) where the trial will be conducted	1 / 5 / 6
Eligibility criteria	14a	Eligibility criteria for participants	5
	14b	If applicable, eligibility criteria for sites and for individuals who will deliver the interventions (e.g., surgeons, physiotherapists)	
Intervention and comparator	15a	Intervention and comparator with sufficient details to allow replication including how, when, and by whom they will be administered. If relevant, where additional materials describing the intervention and comparator (e.g., intervention manual) can be accessed	7-11
	15b	Criteria for discontinuing or modifying allocated intervention/comparator for a trial participant (e.g., drug dose change in response to harms, participant request, or improving/worsening disease)	11
	15c	Strategies to improve adherence to intervention/comparator protocols, if applicable, and any procedures for monitoring adherence (e.g., drug tablet return, sessions attended)	8 / 11
	15d	Concomitant care that is permitted or prohibited during the trial	
Outcomes	16	Primary and secondary outcomes, including the specific measurement variable (e.g., systolic blood pressure), analysis metric (e.g., change from baseline, final value, time to event), method of aggregation (e.g., median, proportion), and time point for each outcome	8 / 9
Harms	17	How harms are defined and will be assessed (e.g., systematically, non-systematically)	11
Participant timeline	18	Time schedule of enrollment, interventions (including any run-ins and washouts), assessments, and visits for participants. A schematic diagram is highly recommended (see Figure)	4

Sample size	19	How sample size was determined, including all assumptions supporting the sample size calculation	5
Recruitment	20	Strategies for achieving adequate participant enrollment to reach target sample size	6
**Methods: Assignment of interventions**			
Randomization:			
Sequence generation	21a	Who will generate the random allocation sequence and the method used	6
	21b	Type of randomization (simple or restricted) and details of any factors for stratification. To reduce predictability of a random sequence, other details of any planned restriction (e.g., blocking) should be provided in a separate document that is unavailable to those who enroll participants or assign interventions	6
Allocation concealment mechanism	22	Mechanism used to implement the random allocation sequence (e.g., central computer/telephone; sequentially numbered, opaque, sealed containers), describing any steps to conceal the sequence until interventions are assigned	6
Implementation	23	Whether the personnel who will enroll and those who will assign participants to the interventions will have access to the random allocation sequence	6
Blinding	24a	Who will be blinded after assignment to interventions (e.g., participants, care providers, outcome assessors, data analysts)	3
	24b	If blinded, how blinding will be achieved and description of the similarity of interventions	3
	24c	If blinded, circumstances under which unblinding is permissible, and procedure for revealing a participant's allocated intervention during the trial	3
**Methods: Data collection, management, and analysis**			
Data collection methods	25a	Plans for assessment and collection of trial data, including any related processes to promote data quality (e.g., duplicate measurements, training of assessors) and a description of trial instruments (e.g., questionnaires, laboratory tests) along with their reliability and validity, if known. Reference to where data collection forms can be accessed, if not in the protocol	8-10
	25b	Plans to promote participant retention and complete follow-up, including list of any outcome data to be collected for participants who discontinue or deviate from intervention protocols	11
Data management	26	Plans for data entry, coding, security, and storage, including any related processes to promote data quality (e.g., double data entry; range checks for data values). Reference to where details of data management procedures can be accessed, if not in the protocol	11

Statistical methods	27a	Statistical methods used to compare groups for primary and secondary outcomes, including harms	12
	27b	Definition of who will be included in each analysis (e.g., all randomized participants), and in which group	12
	27c	How missing data will be handled in the analysis	12
	27d	Methods for any additional analyses (e.g., subgroup and sensitivity analyses)	14
**Methods: Monitoring**			
Data monitoring committee	28a	Composition of data monitoring committee (DMC); summary of its role and reporting structure; statement of whether it is independent from the sponsor and funder; conflicts of interest and reference to where further details about its charter can be found, if not in the protocol. Alternatively, an explanation of why a DMC is not needed	15
	28b	Explanation of any interim analyses and stopping guidelines, including who will have access to these interim results and make the final decision to terminate the trial	
Trial monitoring	29	Frequency and procedures for monitoring trial conduct. If there is no monitoring, give explanation	
**Ethics**			
Research ethics approval	30	Plans for seeking research ethics committee/institutional review board approval	15
Protocol amendments	31	Plans for communicating important protocol modifications to relevant parties	
Consent or assent	32a	Who will obtain informed consent or assent from potential trial participants or authorized proxies, and how	5
	32b	Additional consent provisions for collection and use of participant data and biological specimens in ancillary studies, if applicable	
Confidentiality	33	How personal information about potential and enrolled participants will be collected, shared, and maintained in order to protect confidentiality before, during, and after the trial	10
Ancillary and post-trial care	34	Provisions, if any, for ancillary and post-trial care, and for compensation to those who suffer harm from trial participation	13

*We strongly recommend reading this checklist in conjunction with the SPIRIT 2025 Explanation and Elaboration and the SPIRIT 2025 Expanded Checklist for important clarifications on all the items. We also recommend reading relevant SPIRIT extensions. See www.consort-spirit.org

S2 File. RF Program

<u>Structured Protocol for Second-Generation Intravaginal RF Therapy</u>

S3.1. Device and Technical Specifications

- Device: Capenergy® C500 Urogyne (Capenergy Medical, Barcelona, Spain)
- Type of energy: Capacitive electrical transfer (CET)
- Frequency: The device allows working with 3 frequencies (0.8 MHz, 1 MHz, and 1.2 MHz) that will enable addressing different tissue depths.
- Maximum power: 310 W
- Temperature: The device is equipped with a sensor in the intracavitary electrode that detects the degrees Celsius (°C) reached by the tissue, allowing thermal regulation during the treatment.
- Active electrode: Intravaginal capacitive probe (with single-use probe cover)
- Return electrode: Dispersive plate placed on the lower abdominal region

S3.2. Patient Preparation

1. Explain the procedure to the patient and obtain verbal confirmation of understanding.
2. Ensure the patient has emptied her bladder immediately before the session.
3. Position the patient in dorsal lithotomy.
4. Apply water-soluble gel to the intravaginal probe and cover it with a single-use probe cover.
5. Clean the perineal area with antiseptic solution if necessary.

S3.3. Step-by-Step Application Procedure

1. Place the dispersive return electrode on the lower abdmoninal region and secure it properly.
2. Gently insert the active intravaginal capacitive electrode until it reaches the posterior vaginal fornix.
3. Set the device parameters: Frequency = 1 MHz
4. Begin energy delivery and monitor tissue temperature in real time via the device sensor, aiming to reach 41°C in the target tissues.
5. Once the target temperature of 41 °C is reached and stabilized:
    - Make small up-and-down movements along the urethral canal, dividing the time equally between both sides of the urethra.
6. Total active treatment time per session: 30 minutes.
7. Throughout the session, continuously ask the patient about comfort and heat sensation.

S3.4. Quality Control and Safety Measures

- Tissue temperature must remain between at 41 °C during the entire active phase.
- If the patient reports discomfort ≥ 4/10 on the Visual Analogue Scale (VAS), immediately reduce power or stop the session.
- Session must be stopped immediately in case of:
    - Severe pain or burning sensation
    - Excessive vaginal bleeding or abnormal discharge

- Signs of vaginal trauma or infection
- The physiotherapist must record the following in the session form:
  - Date and session number
  - Actual temperature reached and stability
  - Power used (W)
  - Patient's tolerance (VAS 0–10)
  - Any adverse events or observations

S3.5. Session Recording Form (Example)

Session Number: _____

Date: _______________

Patient Code: ________

Target Temperature: 41 °C Achieved: Yes / No

Duration anterior wall: ______ min   Duration posterior wall: ______ min

Patient tolerance (VAS 0–10): ______

Adverse events: ☐ None ☐ Mild discomfort ☐ Moderate ☐ Severe

Observations / Comments:

Signature of treating physiotherapist: ___________________________

S3.6. Treatment Schedule

- Total sessions: 5
- Frequency: One session per week (every 7 days)
- Total duration of RF treatment: 5 weeks

S3.7. Post-session Instructions

- Advise the patient to report any unusual vaginal discharge, bleeding, pain, or discomfort.
- Normal daily activities may be resumed immediately unless discomfort is present.

S3 File. PFMT Program

Physical Exercise Program Focused on the Pelvic Floor.

The first 4 weeks will include one day of CORE and pelvic floor training in clinic and individual session to ensure understanding of concepts and correct performance of the technique. After this, the therapeutic exercise protocol will be based on proprioception, mobilisation and activation of the structures responsible for the CORE, isometric work, voluntary activation of the CORE muscles, pelvic floor and synergistic muscles such as the gluteus. The work will be both static and dynamic. The exercises will be performed in resisted exhalation and apnoea to facilitate the activation of the lumbo-pelvic complex, as previous programs for patients with abdomino-pelvic dysfunctions have already demonstrated their effectiveness.

<table>
<tr><th>Exercises</th><th>Description</th><th>Intensity and frequency</th><th>Illustrations</th></tr>
<tr><td colspan="4">MONTH 1, Day 1</td></tr>
<tr><td colspan="4">Individual session in a cabin, where anatomical and breathing concepts will be explained and how to carry out the exercises will be explained, checking their correct performance by means of a digital test. The number of sustained and rapid contractions and apnoea time will be determined according to the initial assessment.</td></tr>
<tr><td colspan="4">MONTH 1, Days 2, 4, 6, 8</td></tr>
<tr><td>Pelvic floor and transverse floor activation.</td><td>Face up, legs flexed with heel support and dorsal flexion of the ankle. In resisted exhalation, self-elongation and activation of the transversus abdominis and pelvic floor.</td><td>10 breaths.</td><td>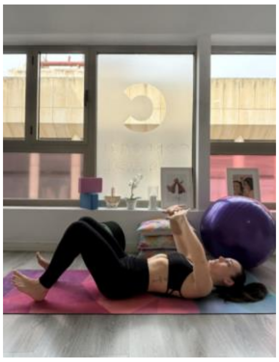</td></tr>
<tr><td>Gluteal bridge.</td><td>Face up, legs flexed with heel support and dorsal flexion of the ankle. In resisted exhalation, self- elongation and activation of the transversus abdominis and pelvic floor together with gluteal elevation (gluteal bridge).</td><td>10 repetitions.</td><td>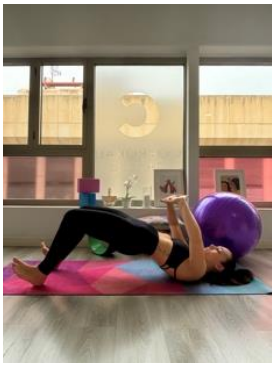</td></tr>
</table>

**Gluteal bridge with alternate leg extension.**	Face up, legs bent with heel support. In resisted exhalation, self-elongation and activation of the transverse and pelvic floor and lifting of the buttocks (gluteal bridge) with unilateral leg stretch. Same exercise with the other leg.	10 repetitions.	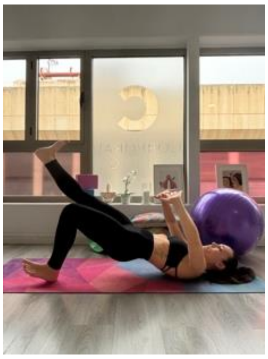
**V-bend abdominals with ball.**	Face up, legs at 90 degrees with ball between them, hip and knee flexion-extension movement is performed with adductor activation. This is performed with exhalation on descent and stretching of the legs.	10 repetitions.	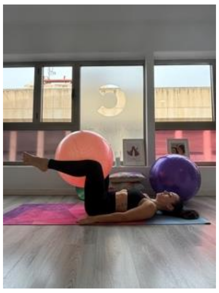 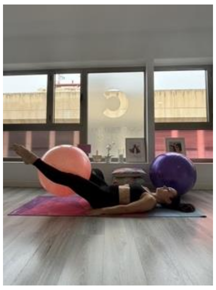
**Leg raise lying on the side.**	On the side with legs straight, activation of the transverse and pelvic floor, perform lifting and lowering movement of the upper leg with maximum amplitude and slow speed and then with minimum amplitude of the movement and maximum speed.	10 repetitions.	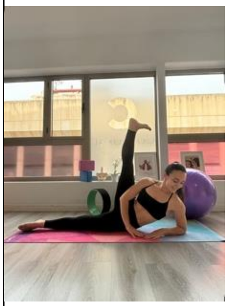 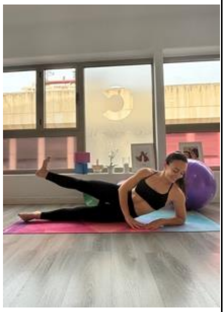
**Leg circling sideways.**	On the side with legs stretched out, activation of the transversus abdominis and awareness of the pelvic floor, perform a circling movement with the upper leg in both directions.	10 repetitions.	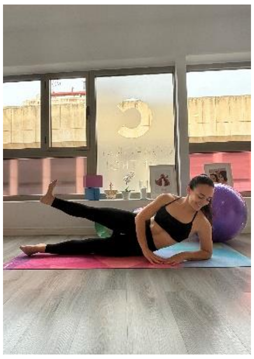

**Side leg flexion and extension.**	On the side, legs aligned with the trunk, activate the transverse and pelvic floor, perform hip and knee flexion and extension movements.	10 repetitions.	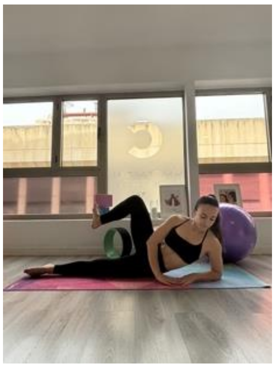
**Sitting contractions on Fitball.**	Sitting on the Fitball, perform pelvic floor contractions with activation of the transversus abdominis, fast and sustained in expiratory time. Do not stay in apnoea.	According to the assessment parameters that we made in consultation.	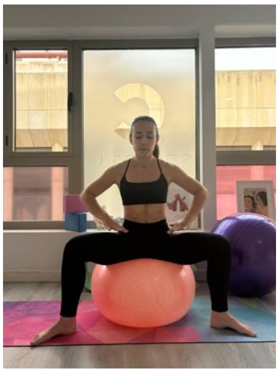
**Pelvic mobility on Fitball.**	On Fitball, awareness and pelvic proprioception movements (perform circles in both directions and infinite circles) to give mobility to the pelvis and relax the CORE and pelvic floor.	60 seconds.	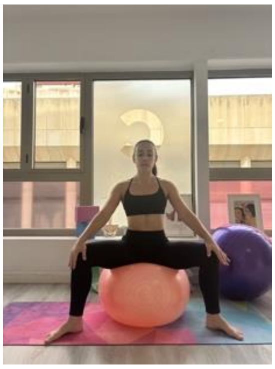
**MONTH 1, Days 3, 5, 7**			
**Pelvic mobility on Fitball.**	On Fitball, awareness and pelvic proprioception movements (perform circles in both directions and infinite circles) to give mobility to the pelvis and relax the CORE and pelvic floor.	60 seconds.	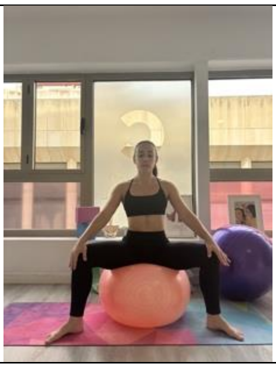

**Pelvic contractions on Fitball.**	Sitting on the Fitball, perform pelvic floor contractions with activation of the transversus abdominis, fast and sustained in expiratory time. Do not stay in apnoea.	According to the assessment parameters that we made in consultation.	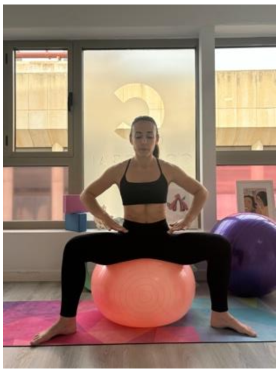
**Self-stretching on Fitball.**	Sitting on the Fitball with heels supported and dorsal flexion of the ankle. After 2 full breaths, stay in apnoea and self-stretch, growing with transverse and pelvic floor activation.	Maintenance of 10sec.	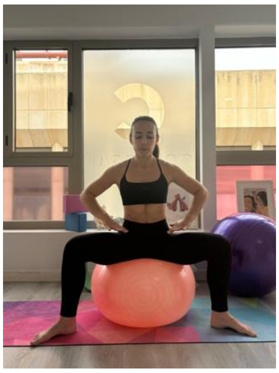
**Self stretching on Fitball with alternating leg lifts.**	Sitting on the Fitball after 2 full breaths, stay in apnoea and self-stretch, growing, with transverse and pelvic floor activation with alternate leg raises.	10 sec. maintenance	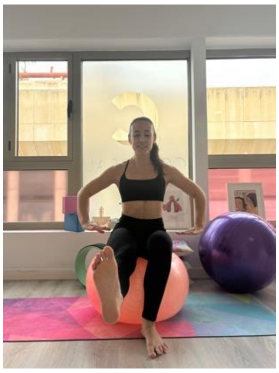
**Quadruped pelvic scales.**	In quadruped movement of pelvic scales with transversus activation and pelvic floor awareness.	10 repetitions.	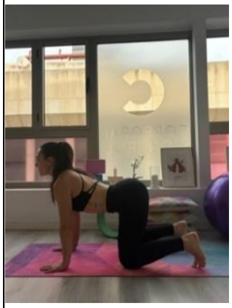 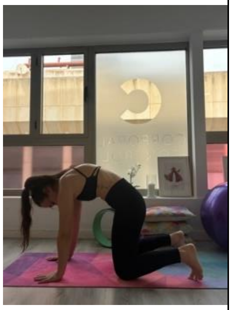

**Lifting of opposite limbs in quadruped.**	In quadruped position, raise one arm and one contralateral leg in resisted expiratory time. Alternate limbs.	10 repetitions.	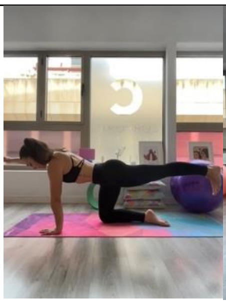 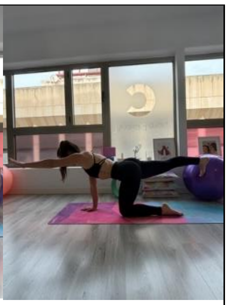
**Hypopressives and self-stretching in quadruped with alternating arm flexion and leg .**	On quadruped, perform hypopressive exercise (after 2 full breaths stay in apnoea, self-elongation and activation of the transverse and pelvic floor and hold for 10 seconds) with flexion of the arms and stretching of one leg. Repeat with the other leg.	Maintenance of 10sec.	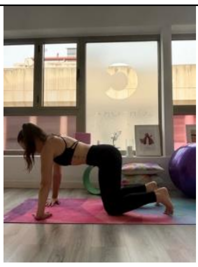 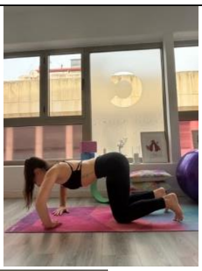 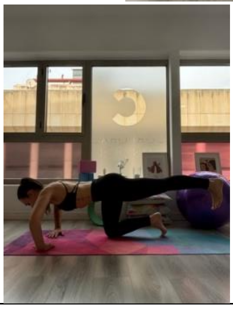
**Mohammedan stance.**	In quadruped position, pelvis mobilisation and dynamic stretching, ending with the posture of the Mohammedan and stretching of the posterior chain. Sit on your heels, rest your head on the floor and stretch your arms in front of you.		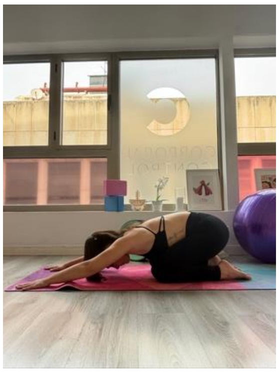

**MONTH 2, Days 9, 11, 13, 15**			
**Pelvic mobility.**	Standing, pelvic awareness exercise. Make infinite circles in both directions with the pelvis, hands on hips.	60 seconds	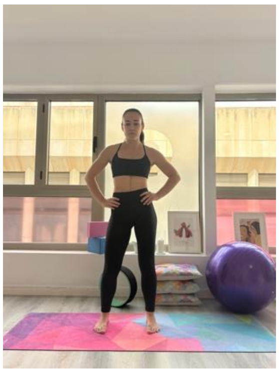
**Pelvic floor contractions.**	Standing, neutral pelvis position, rapid pelvic floor contractions, then held while exhaling. Do not stay in apnoea.	Adapt the number of repetitions according to the assessment made in the consultation.	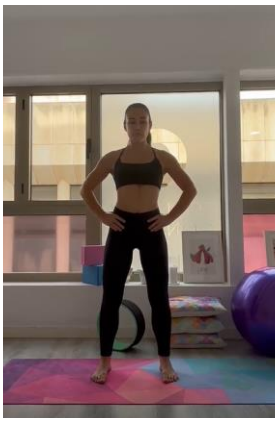
**Balance and motor control on unstable bases.**	Balance and motor control exercises, with conscious breathing on trunk with stable base, on trunk with unstable base and on roller.	Hold each posture for 60 seconds.	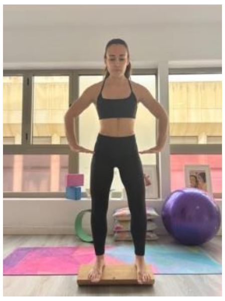 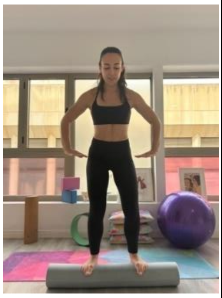
**Deep squats.**	Feet placed wider than hip width apart. Deep squat and raise, activating transverse and pelvic floor.	10 repetitions.	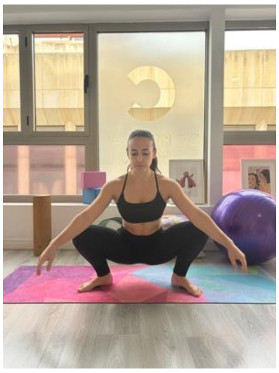

**Isometric squats**	Feet placed wider than hip width apart. Squat held for 3 seconds and raise, all while activating the transverse and pelvic floor.	10 repetitions.	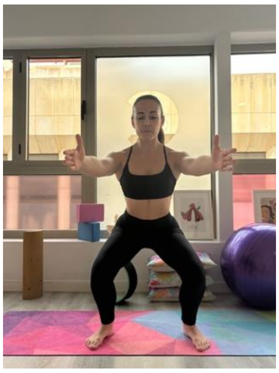
**Alternating strides**	Standing, arms outstretched in front. Forward lunges alternating legs. Activate transverse and pelvic floor.	10 repetitions.	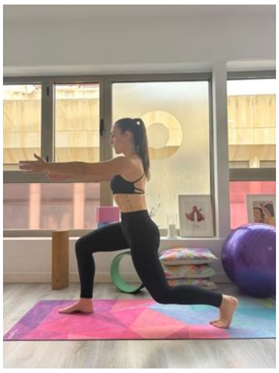
**Self-stretching on the ground.**	Face up, legs flexed with heel support and dorsal flexion of the ankle. In resisted exhalation, self-elongation and activation of the transversus abdominis and pelvic floor.	10 breaths.	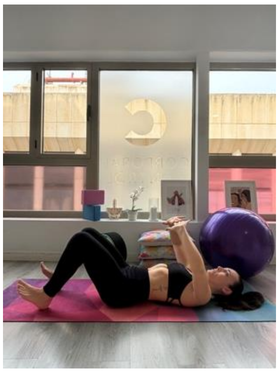
**Gluteal bridge.**	Face up, legs flexed with heel support and dorsal flexion of the ankle. On exhalation resisted self-elongation and activation of the transversus abdominis and pelvic floor together with gluteal lift (gluteal bridge).	10 repetitions.	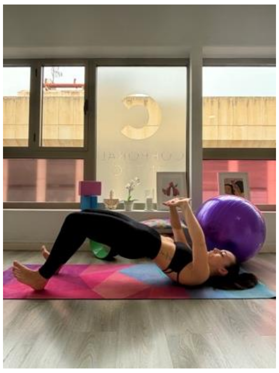

**Gluteal bridge with alternate leg extension.**	Face up, legs bent with heel support. In resisted exhalation, self-elongation and activation of the transversus abdominis and pelvic floor and gluteus lift (gluteus bridge) with unilateral leg stretch. Same exercise with the other leg.	10 repetitions.	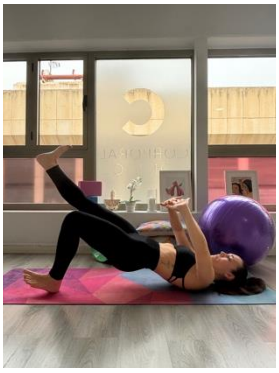
**Activation of adductors with a ball.**	Face up, legs extended at 90° with a ball between them, hip and knee flexion and extension movement with adductor activation. Exercise with normalised breathing.	10 repetitions.	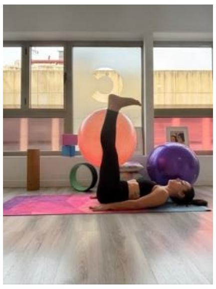 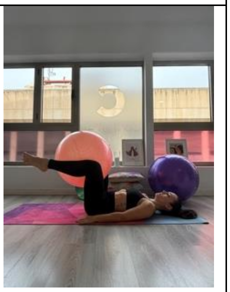
**V-squat alternating bent- leg stretches with ball.**	Face up, legs at 90° with ball between them, hip and knee flexion-extension movement is performed with adductor activation. This is performed with exhalation on descent and stretching of the legs.	10 repetitions.	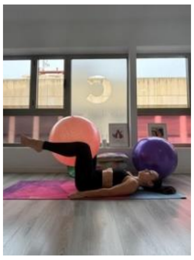 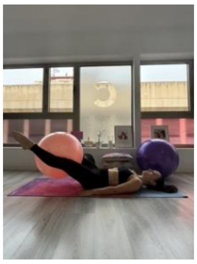
**Active stretching and relaxation and body awareness session.**	Stretching arms, legs, neck, lower back, standing, sitting, calmly, paying attention to your breathing. Return to calm.	5 minutes.	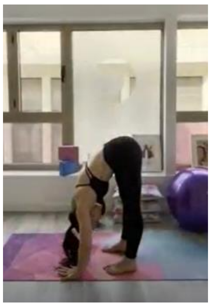 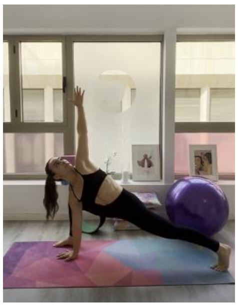 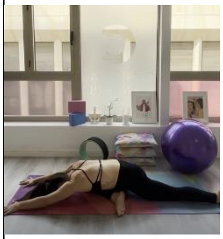 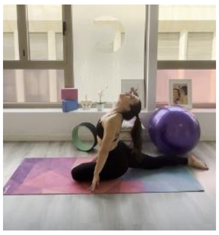

**MONTH 2, Days 10, 12, 12, 14, 16**			
**Awareness and proprioception.**	Face up, awareness exercises and pelvic proprioception, performing sustained and rapid contractions.	According to initial patient assessment.	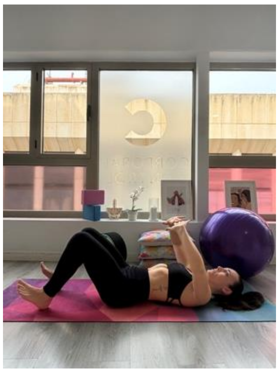
**Self-elongation.**	Face up, legs flexed with heel support and dorsal flexion of the ankle. In resisted exhalation, self- elongation, growing and activation of the transversus abdominis and pelvic floor.	10 breaths.	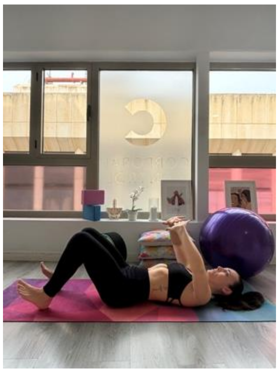
**Gluteal bridge.**	Face up, legs flexed with support of heels and dorsal flexion of the ankle. In resisted exhalation, self- elongation and activation of the transversus abdominis and pelvic floor together with gluteal elevation (gluteal bridge).	10 repetitions.	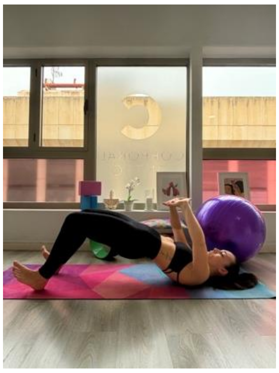
**Gluteal bridge with alternate leg extension.**	Face up, legs bent with heel support. In resisted exhalation, self-elongation and activation of the transversus abdominis and pelvic floor and gluteus lift (gluteus bridge) with unilateral leg stretch. Same exercise with the other leg.	10 repetitions.	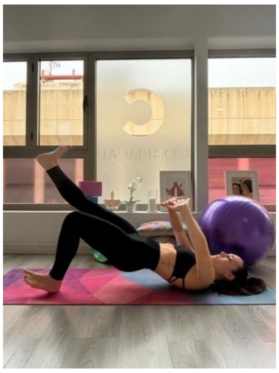

**Leg raise lying on the side.**	On the side with legs straight, activation of the transverse and pelvic floor, perform lifting and lowering movement of the upper leg with maximum amplitude and slow speed and then with minimum amplitude of the movement and maximum speed.	10 repetitions.	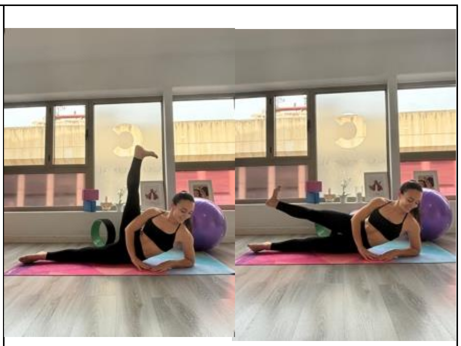
**Circumduction of the leg sideways.**	On the side with legs stretched out, activation of the transversus abdominis and awareness of the pelvic floor, perform a circling movement with the upper leg in both directions.	10 repetitions.	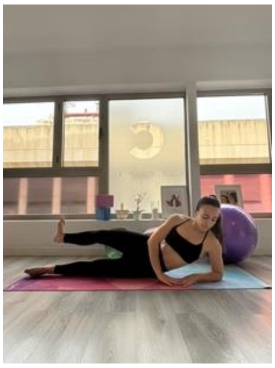
**Side leg flexion and extension.**	On the side, legs aligned with the trunk, activate the transverse and pelvic floor, perform hip and knee flexion and extension movements.	10 repetitions.	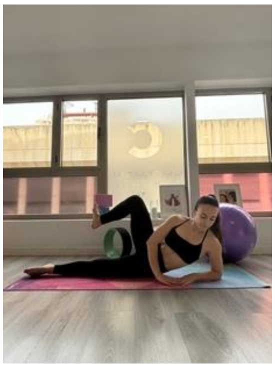
**Hypopressives and self-stretching in inverted dog.**	On the quadruped perform hypopressive exercise (after 2 full breaths, go into apnoea and activate transverse and pelvic floor and hold for 10 seconds) with push-up and leg stretching (mountain or dog yoga position inverted).	10 seconds.	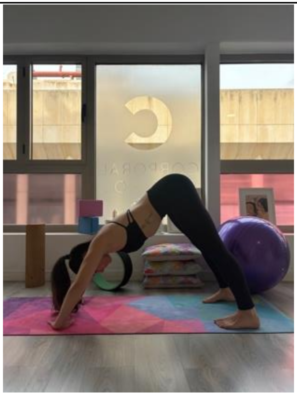

**Hypopressives and self-stretching in inverted dog with alternating leg lifts.**	On the quadruped perform hypopressive exercise (after 2 full breaths, remain in apnoea and activate transversus abdominis and pelvic floor and hold for 10 seconds) with flexion of upper limbs and stretching of the lower limbs (mountain or dog yoga position Inverted) and add one leg lift to the ceiling and hip and knee flexion-extension movement. Repeat with the other leg.	10 seconds.	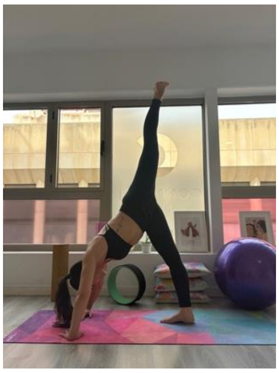
**Front plank with arms bent.**	Front plank on toes and support on forearms. Back and legs aligned, abdominals strong.	60 seconds.	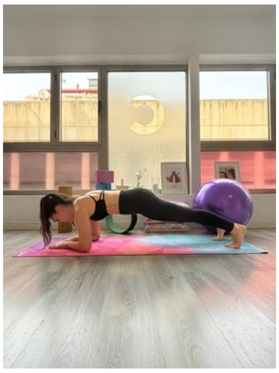
**Front plank with arms outstretched.**	Front plank on toes and support on hands, arms extended. Back and legs aligned. Strong abdominals.	60 seconds.	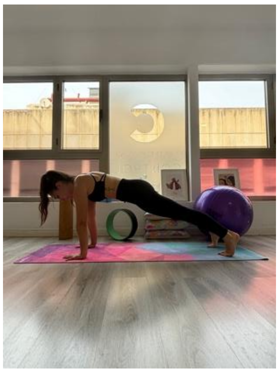
**Side plates.**	Lateral plank, feet together, support on one arm stretched out and the other stretched up. Keep pelvis aligned with trunk. Repeat on the other side.	60 seconds with 30 seconds rest between each.	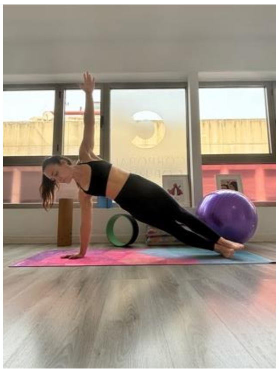

**Front plank with alternating arm and leg raises.**	Front plate with alternating elevation of left arm and right leg and their opponents with strong abdominals and pelvic floor awareness.	10 repetitions (5 on each side).	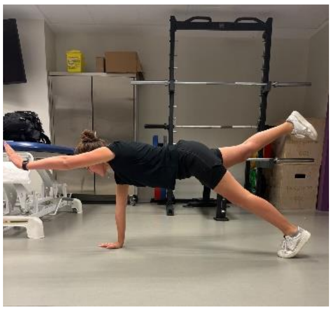
**Front plank - climber.**	Front plank on outstretched arms. Bring the knee to the elbow on the same side, alternating legs.	10 repetitions.	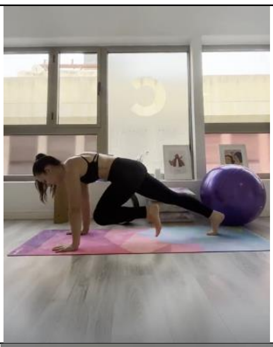
**Exhalation crunch.**	Crunch on exhalation, activating abdominals, head lift with hands behind the head.	10 repetitions.	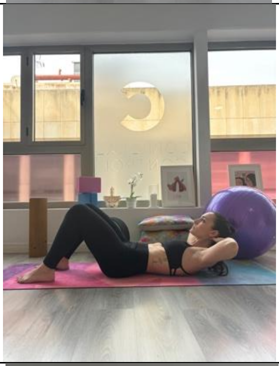
**Exhalation crunch with lateral tilt.**	Exhalation crunch with lateral tilt. Hands crossed behind the head, elbow reaching for the opposite knee.	10 repetitions on each side.	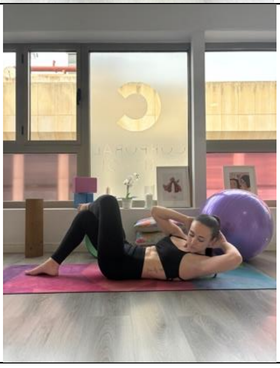
**Crunch with trunk elevation and laterality.**	Legs bent without supporting heels. Trunk upright, contracting abdominals, trunk rolls to each side.	10 repetitions on each side.	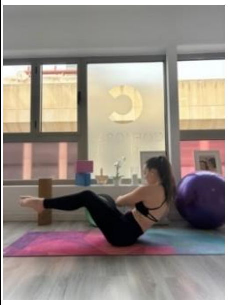 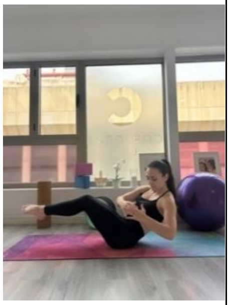

**Oblique.**	Face up, legs bent, resting on heels. Slight elevation of the head and hands reaching for each heel alternately.	10 repetitions on each side.	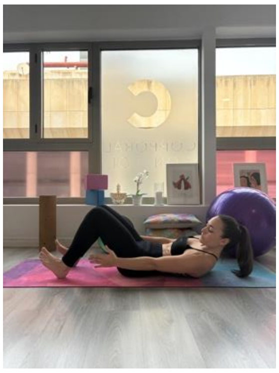
**Active stretching and relaxation and body awareness session.**	Stretching arms, legs, neck, lower back, standing, sitting, calmly, paying attention to your breathing.	5 minutes.	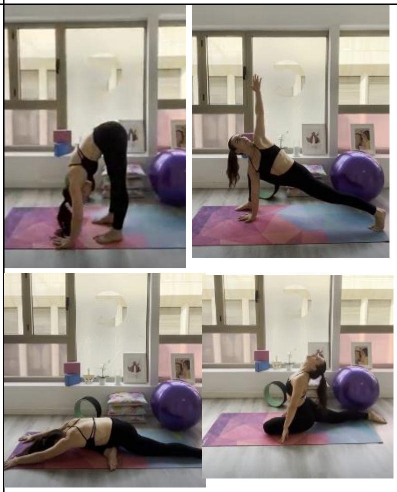

During this 3rd month, we will perform mobility, proprioception and PFMT exercises with increased loading through posture and movement. External load up to 60% of your RM will be introduced according to the progression of loads in terms of strength, endurance and health.

**MONTH 3, Days 17, 19, 21, 23**			
**Gluteal bridge face**	Face up, legs flexed with heel support and dorsal flexion of the ankle. In resisted exhalation, self- elongation and activation of the transversus abdominis and pelvic floor together with gluteal elevation (gluteal bridge).	10 repetitions.	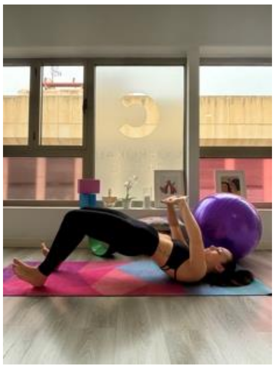

**Gluteal bridge with alternating leg stretching on the back with alternating leg stretching**	Face up, legs bent with heel support. In resisted exhalation, self-elongation and activation of the transversus abdominis and pelvic floor and gluteus lift (gluteus bridge) with unilateral leg stretch. Same exercise with the other leg.	10 repetitions.	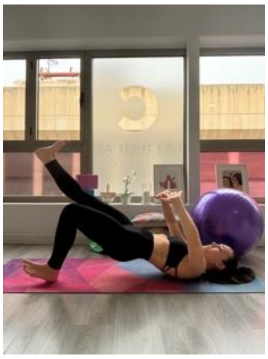
**V-shape sit-ups alternating bent-leg stretches with a ball.**	Face up, legs at 90° with ball between them. Perform hip and knee flexion-extension movement with adductor activation. This is performed with exhalation on the descent and stretching of the legs.	10 repetitions.	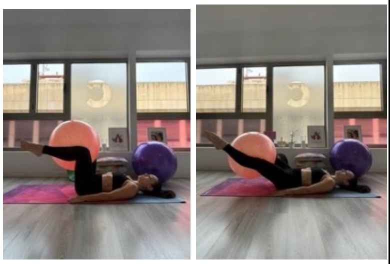
**Front plank with arms bent.**	Front plank on toes and support on forearms. Back and legs aligned, abdominals strong.	90 seconds.<br>Rest: 30 sec	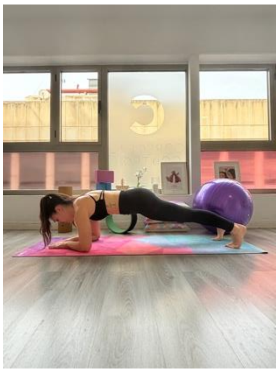
**Front plank with arms outstretched.**	Front plank on toes and support on hands, arms extended. Back and legs aligned. Strong abdominals.	90 seconds. Rest: 30sec.	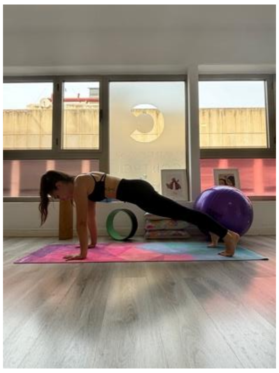

**Lateral plank.**	Lateral plank, feet together, support on one arm stretched out and the other arm stretched up. Repeat on the other side.	90 seconds.<br>Rest: 30	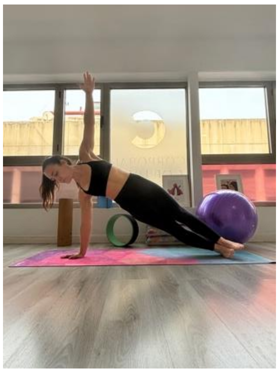
**Front plank with alternating arm and leg raises.**	Front plate with alternating elevation of left arm and right leg and their opposites with strong abdominals and pelvic floor awareness. Repeat with the other side.	10 repetitions (5 on each side).	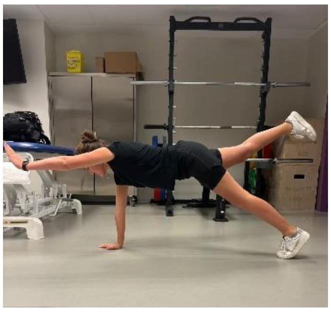
**Front plank - climber.**	Front plank on outstretched arms. Bring the knee to the elbow on the same side,<br>alternating legs.	10 repetitions.	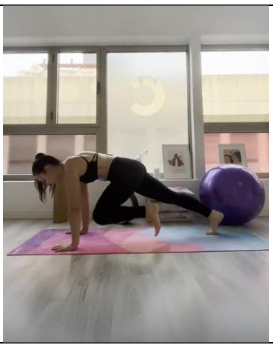
**Active stretching and relaxation and body awareness session.**	Stretching arms, legs, neck, lower back, standing, sitting, calmly, paying attention to your breathing.<br>Return to calm.	5 minutes.	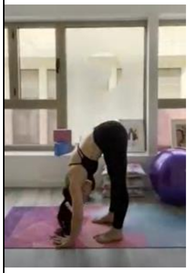 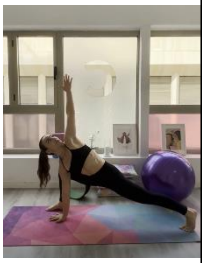 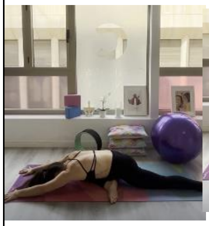 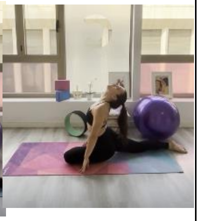

**MONTH 3, Days 18, 20, 20, 22, 24**			
**Pelvic mobility.**	Standing, pelvic awareness exercise. Make infinite circles in both directions with the pelvis, hands on hips.	60 seconds.	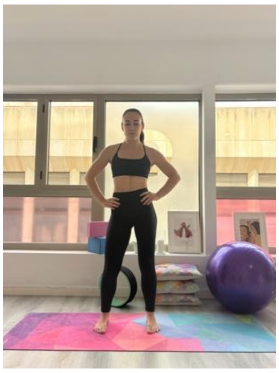
**Pelvic floor contractions.**	Standing, neutral pelvis position, quick pelvic floor contractions, then hold and exhale. Do not stay in apnoea.	Adapt the number of repetitions according to the assessment made in the consultation.	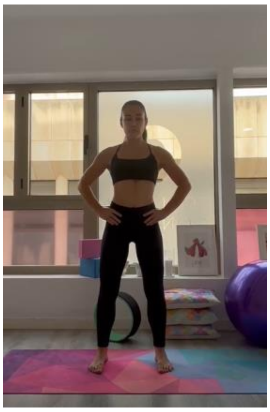
**Balance and motor control on trunk with stable base.**	On the trunk, with the base stable on the floor, do ketellball push-ups and then rotate the ketellball around your abdomen.	10 repetitions of each.	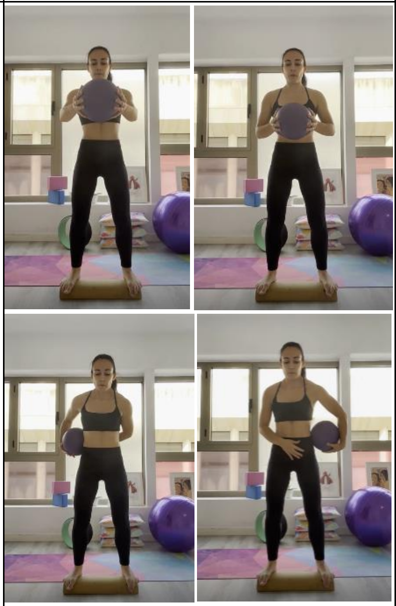

**Balance and motor control on trunk with unstable base.**	On the trunk, with the base unstable on the floor, do ketellball push-ups and then rotate the ketellball around your abdomen.	10 repetitions of each.	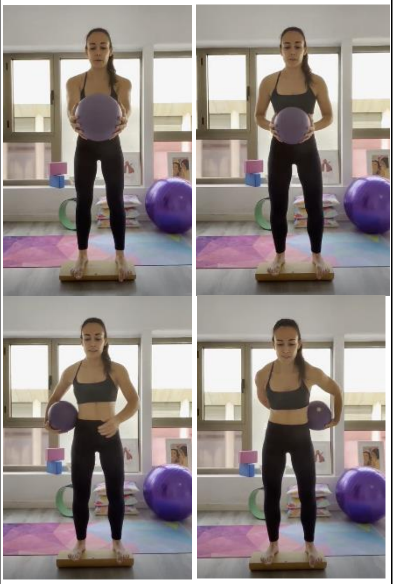
**Balance and motor control on a roller.**	On the roller, do ketell-ball push-ups and then rotate the ketellball around your abdomen.	10 repetitions of each.	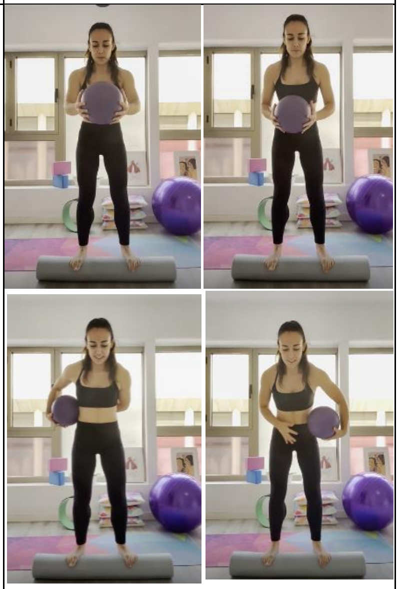
**Deep squat with ketellball.**	Feet placed wider than hip width apart. Hold ketellball with arms extended in front. Deep squat and raise, activating transverse and pelvic floor.	10 repetitions.	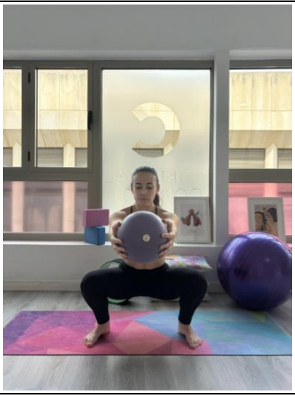

**Isometric squat with ketellball.**	Feet placed wider than hip width apart. Hold ketellball with arms extended in front of you. Deep squat, hold for 3 seconds and raise, all activating transverse and pelvic floor.	10 repetitions.	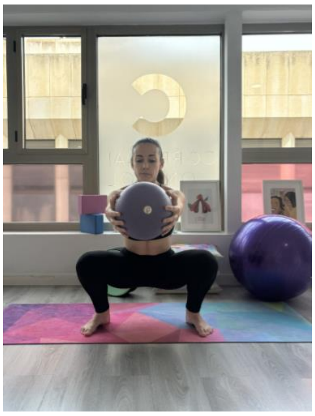
**Alternating stride with**	Standing, catch ketellball with arms extended in front. Lunge forward alternating legs. Activating transverse and pelvic floor.	10 repetitions of each leg.	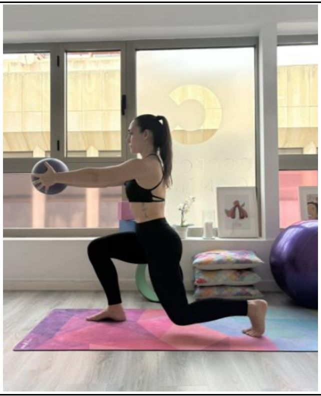
**Hypopressives and self-stretching in inverted dog.**	On the quadruped perform hypopressive exercise (after 2 full breaths, go into apnoea and activate transverse and pelvic floor and hold for 10 seconds) with flexion of upper limbs and stretching of the lower limbs (mountain or dog yoga position inverted).	10 seconds.	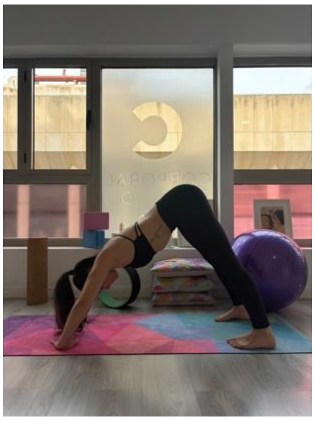
**Hypopressives and self-stretching in inverted dog with alternating leg lifts.**	On the quadruped perform hypopressive exercise (after 2 full breaths, remain in apnoea and activate transversus abdominis and pelvic floor and hold for 10 seconds) with flexion of upper limbs and stretching of the lower limbs	10 seconds with each leg (20 seconds).	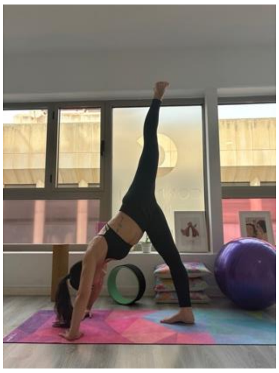

	(mountain or dog yoga position Inverted) and add one leg lift to the ceiling and hip and knee flex- ion- extension move- ment.<br>Repeat with the other leg.		
**Crunch with ketell- ball.**	Lie on your back, sup- port your heels, take the ketellball and raise the trunk a few de- grees, activating the transverse and pelvic floor in expiratory time.	10 repetitions.	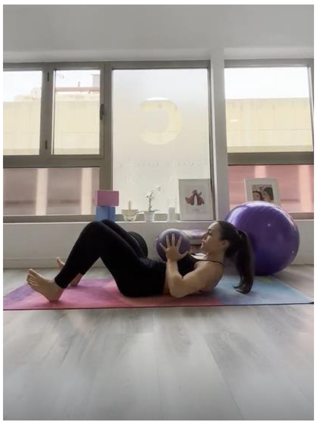
**Crunch with side bends.**	Same exercise, but with 2 ketellballs (1 in each hand) or weights, and instead of raising the trunk, do inclines by bringing the ketellball closer to each heel.	10 repetitions.	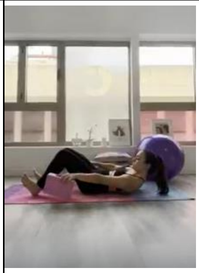 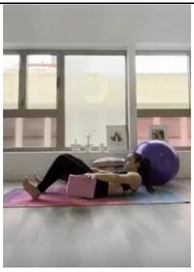
**Crunch with trunk lift and laterality.**	Same position, but place the lateral side of the left foot on the op- posite knee. Lifting the trunk with the ketell- ball, bring the right shoulder to the left knee. Repeat on the other side.	10 repetitions.	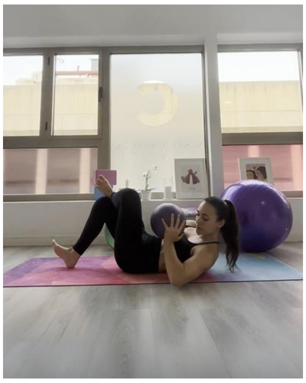
**Oblique with lift and weight.**	Sitting, with the trunk leaning backwards and without supporting the legs, carry the ketellball alternately to the left and right.	10 repetitions on each side.	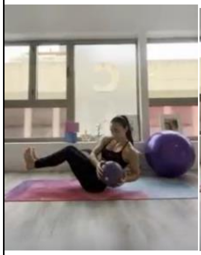 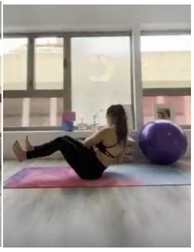

**Active stretching and relaxation and body awareness session.**	Stretching arms, legs, neck, lower back, standing, sitting, calmly, paying attention to your breathing.	5 minutes	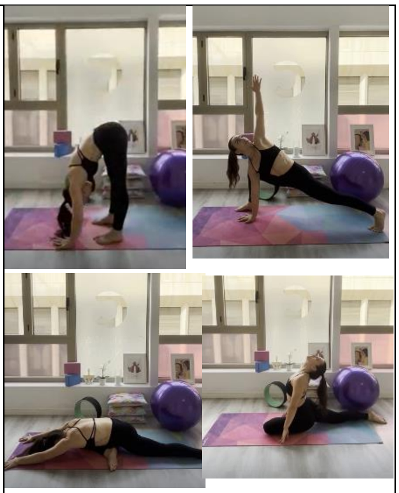

During the 4th month, we will re-evaluate the RM for load management. We will increase it to 75% and focus on working and activating the CORE and pelvic floor in a dynamic way, with normalised breathing and introducing impact and fatigue, which are demonstrable risk factors in pelvic pathology, with the aim of automating the abdominopelvic synergy and its competence in everyday situations, as well as physical exercise.

**MONTH 4, Days 25, 27, 29, 31**			
**Gluteal bridge with alternate leg extension.**	Face up, legs bent with heel support. In resisted exhalation, self-elongation and activation of the transversus abdominis and pelvic floor and gluteus lift (gluteus bridge) with unilateral leg stretch. Same exercise with the other leg.	10 repetitions.	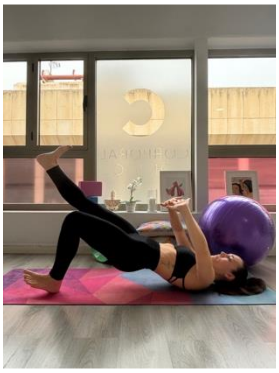
**"Hip thrust with ketellball**	Face up, knees bent with heels on the floor, lifting toes to the ceiling, slowly release the air while contracting the abdomen and pelvic floor and using the ketellball to lift the pelvis.	10 repetitions.	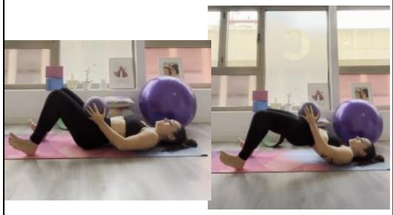

**V-shape sit-ups alternating bent-leg stretches with a ball.**	Face up, legs at 90°° with a ball between them, hip and knee flexion-extension movement is performed with adductor activation. This is performed with exhalation in the descent and stretching of the legs.	10 repetitions.	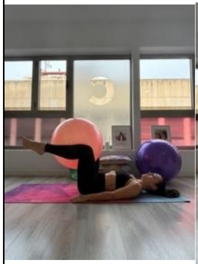 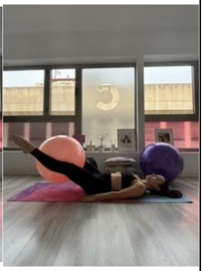
**Abdominals in hip and knee flexion and extension with ball.**	Face up, knees with hips at 90° and a ball between the knees squeezing to activate the inner thigh muscles, bring the feet towards the ceiling and lower the feet to the floor without touching it.	10 repetitions.	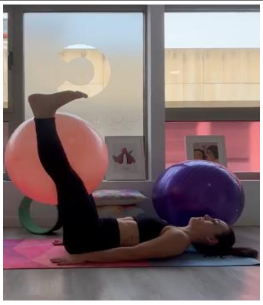 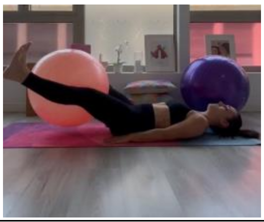
**Front plank with arms bent.**	Front plank on toes and support on forearms. Back and legs aligned, abdominals strong.	90 seconds. Rest : 30s	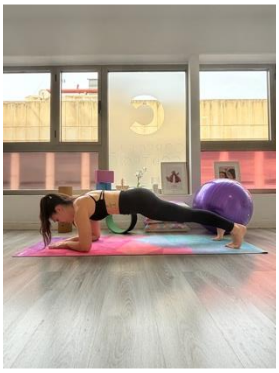
**Front plank with arms outstretched.**	Front plank on toes and support on hands, arms extended. Back and legs aligned. Strong abdominals.	90 seconds. Rest : 30s	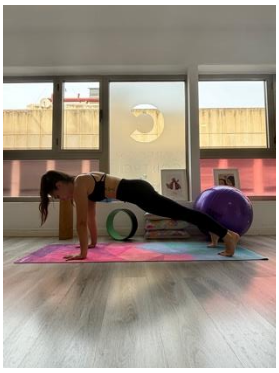

**Side planks**	Lateral plank, feet together, support on one arm stretched out and the other arm stretched up. Repeat on the other side.	60 seconds with 30 seconds rest between each.	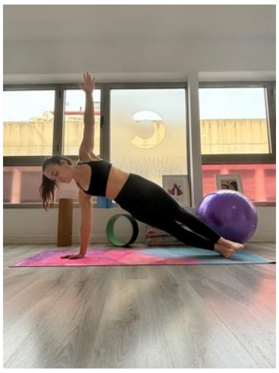
**Front plank with alternating arm and leg raises.**	Front plate with alternating elevation of left arm and right leg and their opposites with strong abdominals and pelvic floor awareness. Repeat with the other side.	10 repetitions (5 on each side).	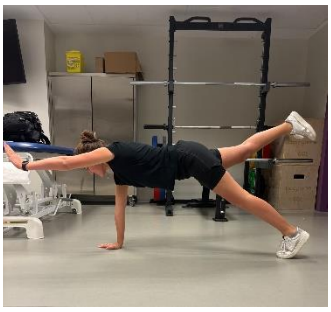
**Front plank - climber.**	Front plank on outstretched arms. Bring the knee to the elbow on the same side, alternating legs.	10 repetitions.	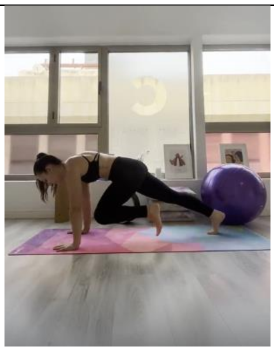
**Hypopressives and self-stretching in inverted dog with alternating leg lifts.**	On the quadruped perform hypopressive exercise (after 2 full breaths, remain in apnoea and activate transversus abdominis and pelvic floor and hold for 10 seconds) with flexion of upper limbs and stretching of the lower limbs (mountain or dog yoga position Inverted) and add one leg lift to the ceiling and hip and knee flexion- extension movement. Repeat with the other leg.	10 seconds.	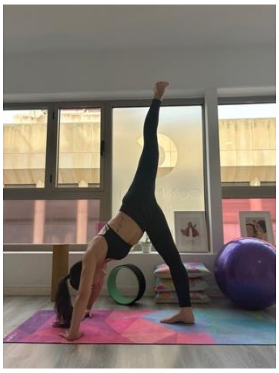

**Active stretching and relaxation and body awareness session.**	Stretching arms, legs, neck, lower back, standing, sitting, calmly, paying attention to your breathing. Return to calm.	5 minutes.	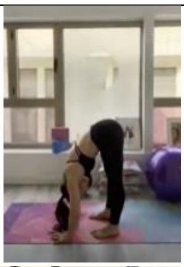 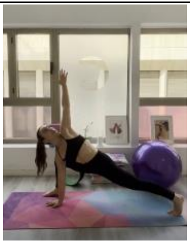 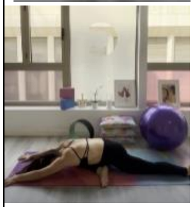 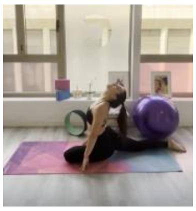

**MONTH 4, Days 26, 28, 30, 32**			
**Pelvis mobility and relaxation of the CORE and pelvic floor**	Standing, pelvic awareness exercise. Make infinite circles in both directions with the pelvis, hands on hips.	60 seconds	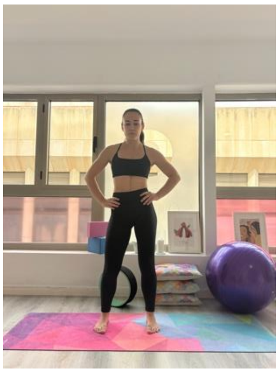
**Ambulation Exercise with Sustained Contractions**	Walking at a comfortable pace while performing fast, sustained contractions, without going apnoeic.	Each person will do a specific number of repetitions according to their assessment.	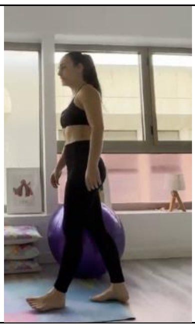
**"Skeeping" with transverse activation and SP awareness**	Lift the knees alternately as if we were running in place, paying special attention to activating the CORE and maintaining awareness of the pelvic floor.	Each person will do a specific number of repetitions according to their assessment.	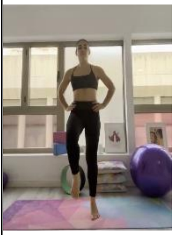 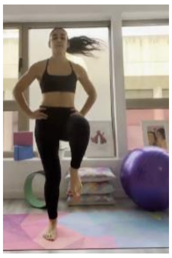

**Balance and motor control exercises on trunk with**	Initially perform this exercise on a stable base, later on a trunk with an unstable base or on a roller. Bring the ketellball between the legs and push the hips forward and bring the ketellball above the chest, lower the ketellball in a controlled manner.	10 repetitions.	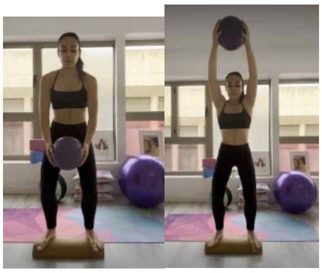
**Isometric squat with ketellball.**	Feet placed wider than hip width apart. Hold ketellball with arms extended in front of you. Deep squat, hold for 3 seconds and raise, all activating transverse and pelvic floor.	10 repetitions.	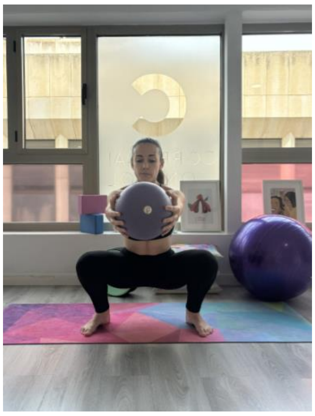
**Deep squat with ketellball**	Feet placed wider than hip width apart. Pick up ketellball with arms extended in front. Deep squat and pull up, all activating transverse and pelvic floor.	10 repetitions.	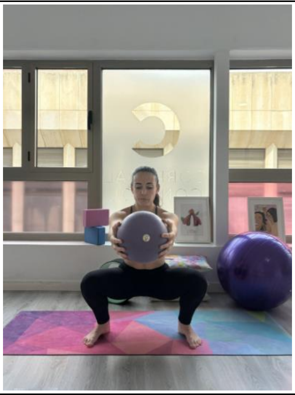
**Alternating stride with**	Standing, catch ketellball with arms extended in front. Lunge forward alternating legs. Activating transverse and pelvic floor.	10 repetitions of each leg.	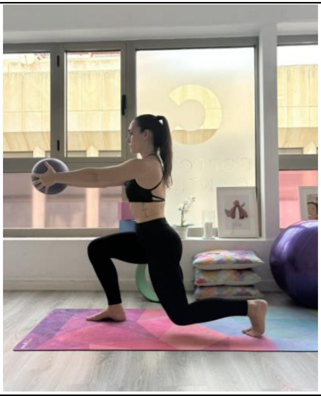

**Burpees**	Standing, with feet shoulder width apart and arms at your sides, activate the transverse abdominis by drawing the navel in and up and contracting the pelvic floor muscles at the same time. From the standing position, lower into a squat position and place hands on the floor, jump up and bring feet into plank position, hold plank position and return to start.	10 repetitions.	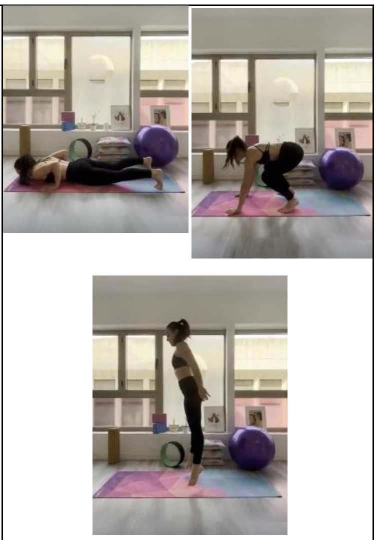
**Jump into the drawer**	Feet together, jump over a crate and jump down. It can be done like climbing stairs. Raise awareness of pelvic floor activation.	Repetitions: 10	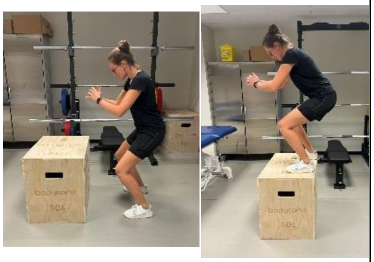
**Exhalation crunch with ketellball**	Crunch on exhalation, activating abdominals, head lift with hands behind the head.	10 repetitions.	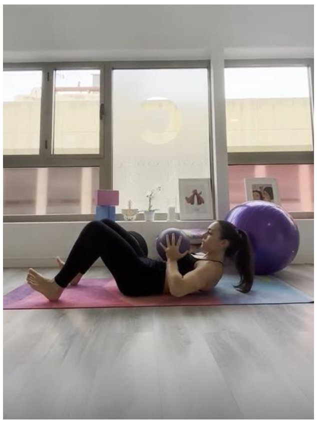

**Crunch with side bends.**	Same exercise, but with 2 ketellballs (1 in each hand) or weights, slight flexion of the head, and instead of raising the trunk, do inclines bringing the ketellball close to each heel.	10 repetitions.	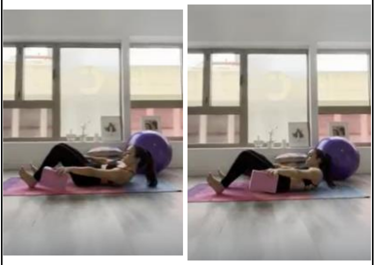
**Crunch with trunk lift and laterality.**	Same position, but place the lateral side of the left foot on the opposite knee. Lifting the trunk with the ketellball, bring the right shoulder to the left knee. Repeat on the other side.	10 repetitions.	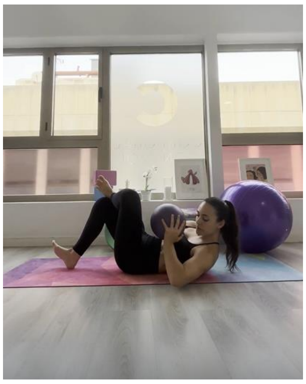
**Oblique with lift and weight.**	Sitting, with the trunk leaning backwards and without supporting the legs, carry the ketellball alternately to the left and right.	10 repetitions on each side.	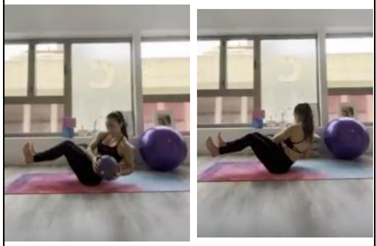
**Active stretching and relaxation and body awareness session.**	Stretching arms, legs, neck, lower back, standing, sitting, calmly, paying attention to your breathing. Return to calm.	5 minutes.	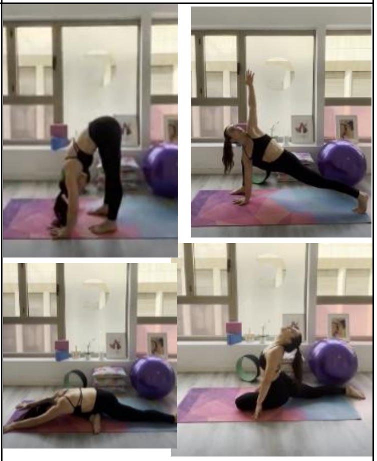
